# Prevalence and risk factors of negative emotions in infertile women: a systematic review and meta-analysis

**DOI:** 10.3389/fpubh.2025.1701381

**Published:** 2025-12-04

**Authors:** Liuxin Hu, Youchang Yuan, Yanjing Li, Mian Cai, Jie Yin, Lijuan Zhu

**Affiliations:** 1 Department of Reproductive Medicine, Jiangxi Province Hospital of Integrated Chinese and Western Medicine, Nanchang, China; 2 Graduate School, Jiangxi University of Chinese Medicine, Nanchang, China; 3 College of Nursing, Jiangxi University of Chinese Medicine, Nanchang, Jiangxi, China

**Keywords:** female infertility, anxiety, depression, related factors, prevalence, systematic review, meta-analysis

## Abstract

**Objective:**

The purpose of this study was to investigate the prevalence of anxiety and depression in female infertility patients, as well as the risk factors that are linked to these conditions, through a systematic review and meta-analysis.

**Methods:**

Eight databases—PubMed, Web of Science, Embase, Cochrane Library, Sinomed, CNKI, VIP, and WanFang—were thoroughly searched, with the search period lasting until February 28, 2025. Research on anxiety or depression and the risk factors linked to it that involved female infertility patients was included. Information was taken from each study, including study region, patient age, prevalence rates, and risk factors for anxiety or depression in female infertility patients.

**Results:**

It was discovered that 41% [95% CI (0.35, 0.47)] of female infertility patients had anxiety, and 42% [95% CI (0.36, 0.48)] had depression. Age [OR = 1.38, 95% CI (1.10, 1.73)], duration of infertility [OR = 1.68, 95% CI (1.30, 2.17)], treatment expenses [OR = 2.04, 95% CI (1.78, 2.34)], and lack of knowledge about Assisted Reproductive Technology (ART)-related Information [OR = 1.70, 95% CI (1.26, 2.29)] were significantly associated with anxiety. Age [OR = 1.16, 95% CI (1.00, 1.33)], duration of infertility [OR = 1.83, 95% CI (1.56, 2.16)], treatment expenses [OR = 1.47, 95% CI (1.25, 1.74)], history of miscarriage [OR = 2.17, 95% CI (1.43, 3.31)], and primary infertility [OR = 2.15, 95% CI (1.55, 3.00)] were risk factors for depression. Other factors analyzed, such as place of residence, no reproductive history, and family income, were not found to be statistically significant.

**Conclusion:**

Women with infertility face a high incidence of anxiety and depression, which is related to factors such as treatment costs, age, and duration of infertility. A history of miscarriage or primary infertility further increases the risk of depression, while a lack of knowledge of assisted reproductive technologies increases the risk of anxiety.

**Systematic review registration:**

PROSPERO, https://www.crd.york.ac.uk/PROSPERO/view/CRD420251036068.

## 1 Introduction

Failure to conceive after 12 months of unprotected intercourse is defined as infertility (1), divided into primary infertility with no previous pregnancy and secondary infertility after prior conception. This condition affects approximately 16.5 to 17.8% of adults worldwide (2) and is a serious problem recognized by the World Health Organization. Limited attention to infertility in many countries leads to serious psychological problems at the individual and societal levels, with statistics showing that 25 to 60% of people with infertility suffer from mental illness (3). The high demand for treatment, combined with pressure from partners, families, and society, makes infertility patients more vulnerable to psychological distress. This stress often produces negative emotional states, including depression, anxiety, stigma, decreased self-esteem, and pessimism, with depression and anxiety being the most common manifestations (4). Studies have shown that female infertility patients often experience more severe anxiety and hopelessness than male patients (5). In addition, untreated depression and anxiety disorders can affect the pregnancy process. The hypothalamic–pituitary-ovarian (HPO) axis can be disrupted by negative emotions, leading to endocrine abnormalities and directly affecting ovarian function. Women experiencing psychological stress may also experience a range of stress responses, including increased catecholamine release and activation of the sympathetic nervous system. This eventually causes excessive contraction of the smooth muscles of the uterus, which prevents the embryo from implanting (6). Therefore, it is essential to understand the frequency and contributing factors of negative emotions in female infertility patients in order to develop targeted treatments that improve patients’ quality of life and potentially improve infertility treatment outcomes.

Researchers have recently focused more on mental health problems experienced by women with infertility, particularly anxiety and depression, due to the rising prevalence of infertility (4, 7). However, most recent studies have been cross-sectional, with only a few meta-analyses (8, 9) addressing the incidence of depression or anxiety in female infertility patients, and one study (10) looking at the prevalence and influencing variables of infertility. Therefore, in order to investigate the incidence of anxiety and depression in female infertility patients and the risk factors associated with them, this study aims to conduct an updated systematic review and meta-analysis based on the latest evidence in Chinese and English literature, with a special focus on the comprehensive assessment of multiple risk factor categories.

## 2 Methods

This review was conducted following the Preferred Reporting Items for Systematic Reviews and Meta-Analyses (PRISMA) checklist (11). The International Prospective Registry for Systematic Reviews (PROSPERO) has registered it under registration number CRD420251036068, and can be accessed via the following URL: https://www.crd.york.ac.uk/PROSPERO/view/CRD420251036068.

### 2.1 Literature search

A systematic search was carried out on eight electronic databases, including PubMed, Web of Science, Embase, Cochrane Library, Sinomed, CNKI, VIP and Wanfang, and medical subject headings were combined with free text terms such as “infertility,” “anxiety,” “depression” and “prevalence” to determine the prevalence of anxiety and depression in infertile women and their influencing factors. The search covered publications in English or Chinese from the database’s inception to February 28, 2025, using PubMed as an example, and Supplementary Table S1 details the full strategy.

### 2.2 Literature inclusion and exclusion criteria

#### 2.2.1 Inclusion criteria

Study population: Female infertility patients, excluding individuals with comorbid physical illness or depression/anxiety due to non-physical causes.

Measurement tools: Validated methods, including structured clinical interviews or standardized scales.

Research content: The prevalence of anxiety or depression in infertile women, the duration of infertility, social support, and other related influencing factors.

Study type: case–control study with well-defined groups and cross-sectional study with simultaneous data collection.

#### 2.2.2 Exclusion criteria

Exclusion criteria include inability to access full-text or basic data, duplicate publications, literature in languages other than Chinese or English, and specific publication types such as reviews, meta-analyses, lectures, and conference abstracts.

### 2.3 Literature screening and information extraction

Two researchers (HLX and YYC) independently screened the literature through a two-step process, including initial title and abstract eligibility assessments, followed by a full-text review, and any discrepancies were resolved in consultation with the third investigator (ZLJ) to reach consensus.

### 2.4 Data extraction

Two uniformly trained researchers (HLX and YYC) independently extracted data using a standardized Excel tool and subsequently cross-validated, resolving uncertainties through a joint original literature review and consulting a third researcher (ZLJ) on unresolved disputes. The extracted data included the first author, year of publication, study area, age of patients, sample size, anxiety/depression detection rate, and influencing factors.

### 2.5 Risk of bias assessment for included studies

The Agency for Healthcare Research and Quality (AHRQ) (12) recommended scale, which has 11 questions with a total score range of 0–11 points, was used to evaluate the quality of cross-sectional studies. The evaluation of each issue involved choosing “yes,” “no,” or “unclear,” with a “yes” response worth one point. Low-quality studies (0–3 points), medium-quality studies (4–7 points), and high-quality studies (8–11 points) were then classified according to the studies’ overall score. The Newcastle-Ottawa Scale (NOS) (13), which consists of eight questions divided into three sections—exposure, comparability, and study population selection—was used to assess case–control studies. Low quality was denoted by a score of ≤ 4 points, medium quality by 5 or 6 points, and good quality by 7–9 points.

### 2.6 Data analysis

Stata 16.0 and RevMan 5.4 were used for statistical analysis. By taking the total number of female infertility patients in each included study and the actual number of cases of anxiety or depression in these patients, we were able to determine the prevalence. To assess the associated risk factors, adjusted odds ratios (OR) with 95% confidence intervals (CI) were also gathered. The I^2^ statistic was used to test for heterogeneity; if I^2^ < 50% and *p* < 0.05, homogeneity was indicated, and a fixed-effect model was used. If not, a random-effects model was selected. The origins of variability were investigated using subgroup analyses, which were visualized using forest plots. We divided the risk factors into four categories for comparison’s sake: patient, disease, therapy, and economic circumstances.

### 2.7 Standardization of psychological measurement tools

The included studies assessed anxiety and depression using multiple psychometric tools, including HADS, DASS-21, SAS, SDS, PHQ-9, and BDI. To ensure comparability across studies with differing scoring systems and clinical thresholds, all studies applied standardized cut-off values established in original validation studies, with detailed comparisons provided in Supplementary Table S4.

## 3 Results

### 3.1 Literature search results

**Figure 1:**
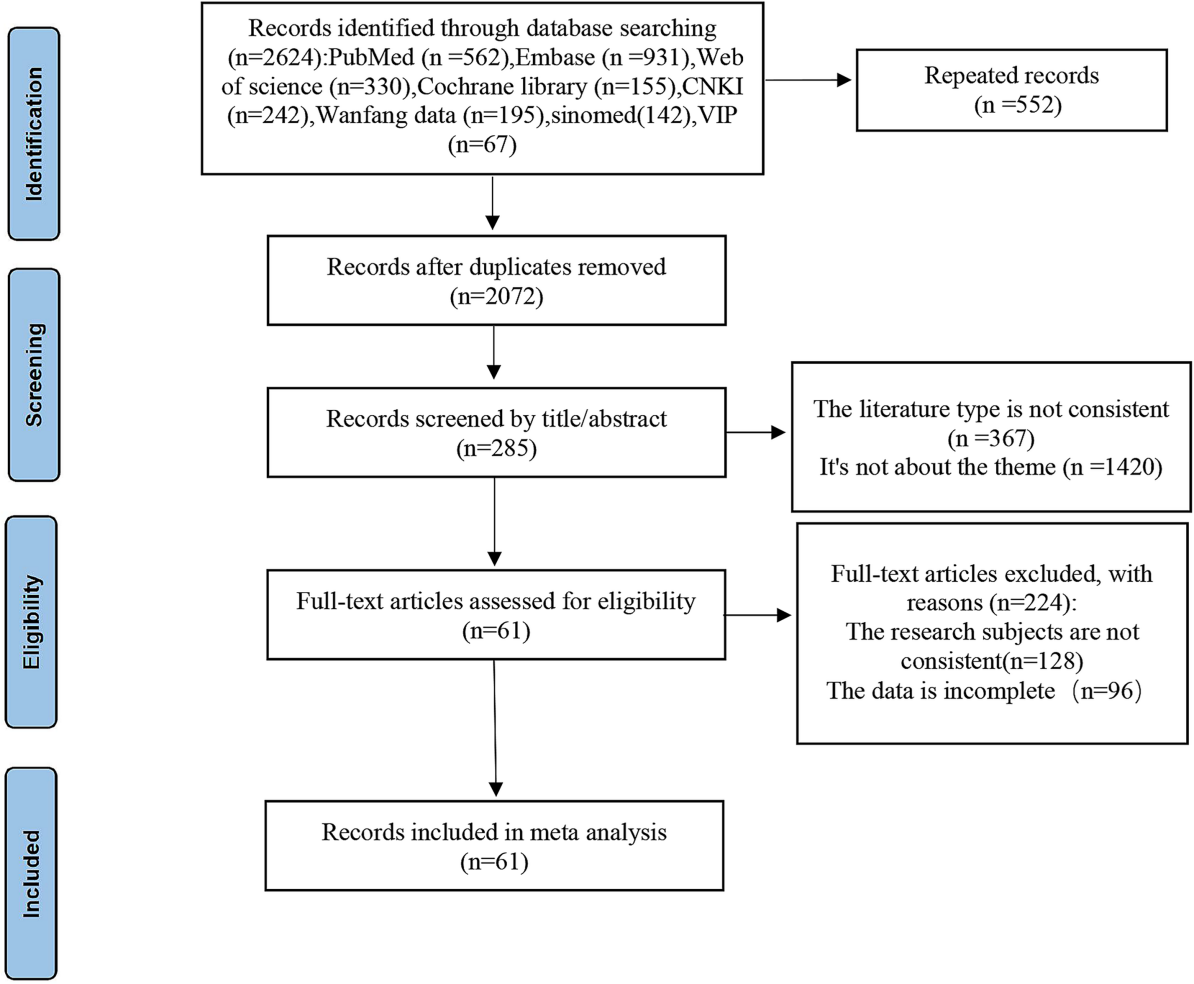
PRISMA flow diagram of the study selection process. *Consider, if feasible to do so, reporting the number of records identified from each database or register searched (rather than total number across all database/registers). **If automation tools were used, indicate how many records were excluded by a human and how many records were excluded by automation tools. Source: Pahe et al. (11). This work is licensed under CC BY 4.0. To view a copy of this license, visit https://creativecommons.org/license/by/4.0/.

Database searches initially identified 2,624 publications, with 2,072 records remaining after duplicate removal, proceeding to title and abstract screening. This excluded 1,787 articles, leading to a full-text review of 285 studies, where 224 were excluded. Ultimately, 61 articles met all inclusion criteria and were included in the meta-analysis. The literature screening process is shown in Figure 1.

### 3.2 Basic characteristics and risk of bias assessment of included literature

**Figure 2:**
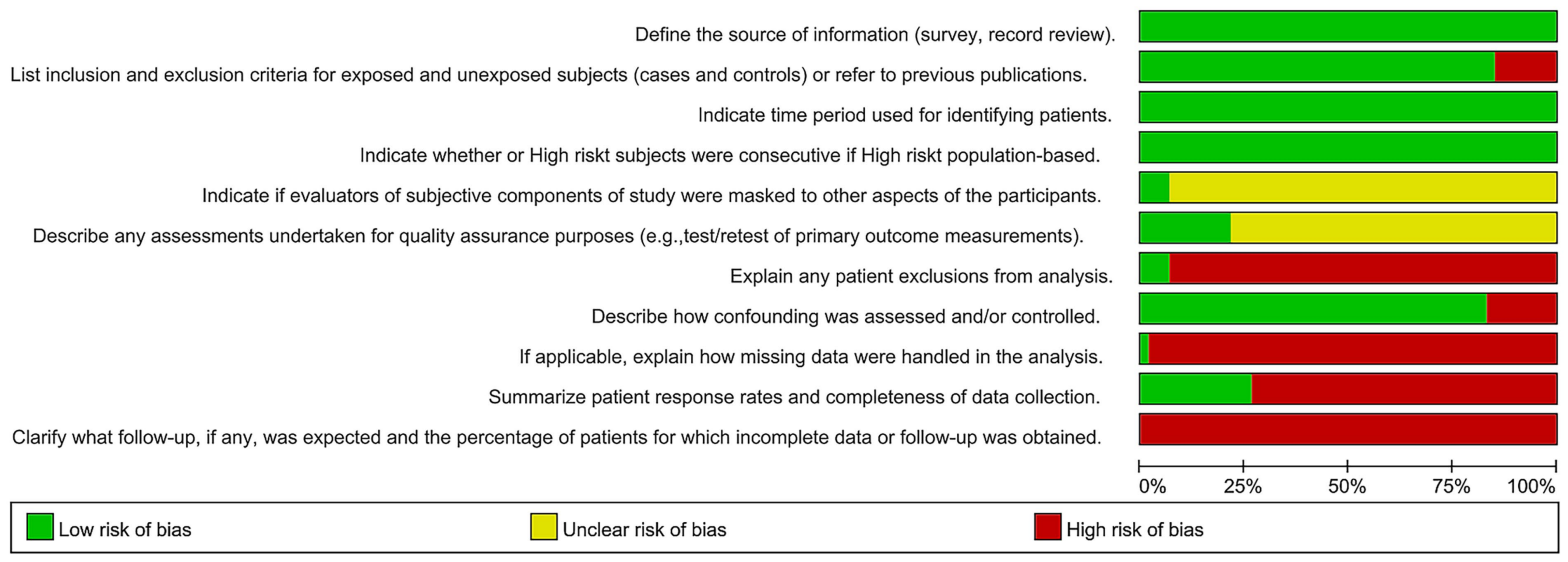
Quality assessment of cross-sectional studies.

**Figure 3:**
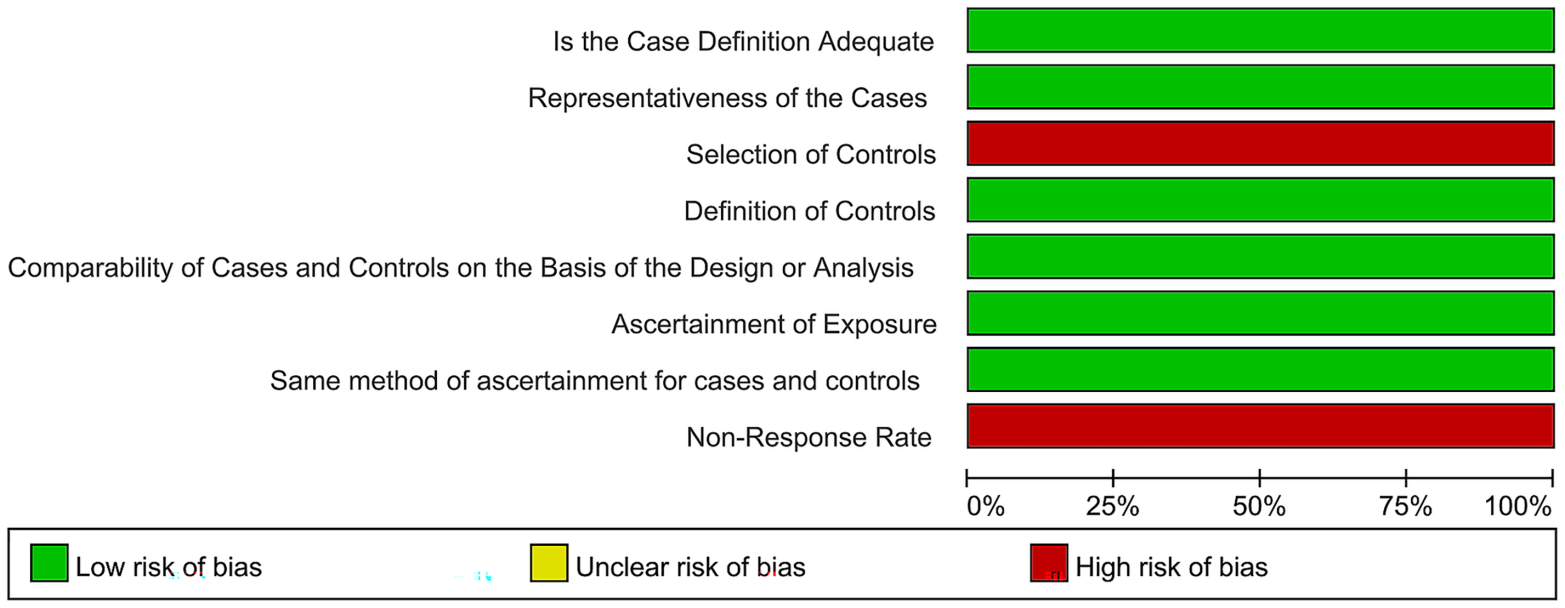
Quality assessment of case–control studies.

This study included a total of 61 articles, encompassing 148,670 patients, with 33 Chinese articles and 28 English articles. Among these, 60 were cross-sectional studies and 1 was a case–control study, with specific characteristics provided in Table 1. Literature quality assessment was conducted using the AHRQ and NOS scales. The results showed that among the cross-sectional studies, 3 articles (14–16) were classified as high-quality studies, 53 articles (17–69) were medium-quality studies, and 4 articles (70–73) were low-quality studies. The only case–control study (74) was rated as high quality. Quality assessment charts are shown in Figures 2, 3.

**Table 1:** Basic characteristics, prevalence of anxiety/depression, and quality assessment of included studies.

Author/year	Country	Total	Study Design	The method of assessment (anxiety/depression)	Events Anxiety	Prevalence Anxiety	Events Depression	Prevalence Depression	Score
Wang, 2014 (17)	China	120	Cross-Sectional Study	HADS/HADS	39	32.5	37	30.83	5
Sun, 2021 (18)	China	125	Cross-Sectional Study	SCL-90/SCL-90	75	60	62	49.6	7
Fang, 2015 (61)	China	207	Cross-Sectional Study	SAS/SDS	34	16.4	90	43.5	4
Wang, 2015 (60)	China	358	Cross-Sectional Study	SAS/SDS	90	25.14	128	35.75	7
Men, 2020 (48)	China	205	Cross-Sectional Study	SAS/SDS	39	19.02	41	20	5
Peng, 2019 (49)	China	297	Cross-Sectional Study	SAS/SDS	44	14.8	64	21.5	7
Cheng, 2011 (66)	China	120	Cross-Sectional Study	NA/BDI	NA	NA	57	47.5	7
Zhang, 2013 (15)	China	137	Cross-Sectional Study	SAS/SDS	99	72.3	78	56.9	8
Wu, 2015 (59)	China	306	Cross-Sectional Study	SAS/NA	188	61.4	NA	NA	4
Chen, 2016 (58)	China	120	Cross-Sectional Study	SAS/SDS	31	25.83	49	40.83	7
He, 2014 (62)	China	100	Cross-Sectional Study	SAS/SDS	44	44	45	0.45	5
Chen, 2013 (64)	China	144	Cross-Sectional Study	SAS/SDS	36	25	30	20.83	6
Zhao, 2018 (50)	China	169	Cross-Sectional Study	SAS/SDS	112	55.9	72	42.3	7
Zhou, 2005 (68)	China	64	Cross-Sectional Study	HAD/HAD	16	25	37	57.81	4
Liu, 2010 (70)	China	211	Cross-Sectional Study	SAS/SDS	87	41.23	165	78.2	3
Hao, 2017 (55)	China	120	Cross-Sectional Study	SAS/SDS	103	85.83	101	84.17	4
Hu, 2021 (46)	China	203	Cross-Sectional Study	SAS/SDS	73	36	67	33	6
Ma, 2022 (45)	China	552	Cross-Sectional Study	SAS/SDS	210	38	181	32.79	5
Liang, 2018 (51)	China	279	Cross-Sectional Study	SAS/SDS	93	32.2	68	24.4	7
Li, 2016 (57)	China	60	Cross-Sectional Study	SAS/SDS	31	51.67	34	56.67	5
Han, 2013 (63)	China	200	Cross-Sectional Study	SAS/SDS	154	77	113	56.5	5
Qi, 2007 (71)	China	213	Cross-Sectional Study	HADS/HADS	69	35.5	52	23.1	3
Hu, 2017 (54)	China	205	Cross-Sectional Study	SAS/SDS	29	14.15	48	23.41	6
Deng, 2018 (52)	China	210	Cross-Sectional Study	HADS/HADS	81	38.6	83	39.5	5
Tan, 2023 (43)	China	110	Cross-Sectional Study	SAS/SDS	34	30.9	35	31.8	5
Cai, 2024 (42)	China	152	Cross-Sectional Study	SAS/SDS	47	30.9	59	38.8	5
Zhu, 2022 (44)	China	90	Cross-Sectional Study	SAS/SDS	40	44.44	34	37.78	7
Li, 2012 (65)	China	538	Cross-Sectional Study	SAS/SDS	207	38.5	66	12.3	5
Wu, 2008 (67)	China	190	Cross-Sectional Study	SAS/CES-D	35	15.6	72	32	4
Wang, 2020 (47)	China	652	Cross-Sectional Study	SAS/SDS	93	14.2	195	29.9	5
Guo, 2017 (56)	China	188	Cross-Sectional Study	SAS/SDS	163	86. 70	87	46. 28	5
Chen, 2019 (14)	China	160	Cross-Sectional Study	SAS/NA	104	65	NA	NA	8
Zhang, 2017 (53)	China	202	Cross-Sectional Study	SAS/SDS	98	48.5	110	56.9	7
Li, 2017 (36)	China	211	Cross-Sectional Study	NA/SDS	NA	NA	107	50.71	5
Ramezanzadeh, 2004 (69)	Iran	370	Cross-Sectional Study	Cattle INAveNAtory/BDI	151	40.8	321	86.8	4
Alhassan, 2014 (39)	Ghana	100	Cross-Sectional Study	NA/BDI	NA	NA	62	62	5
Kumar, 2023 (22)	Indian	160	Cross-Sectional Study	HADS/HADS	83	51.9	69	43.1	4
Lakatos, 2017 (35)	Hungary	225	Cross-Sectional Study	STAI-T/BDI	89	39.6	101	44.8	6
Joelsson, 2017 (34)	Sweden	468	Cross-Sectional Study	HADS/HADS	270	57.6	73	15.7	5
Sulyman, 2019 (32)	Nigeria	207	Cross-Sectional Study	HADS/HADS	57	27.5	53	25.6	5
Anh, 2023 (24)	Vietnam	385	Cross-Sectional Study	DASS 21/DASS 21	178	46.2	68	17.6	4
Adelosoye, 2020 (16)	Nigeria	341	Cross-Sectional Study	NA/SDS	NA	NA	145	42.5	8
Rufai, 2022 (28)	Nigeria	415	Cross-Sectional Study	NA/BDI	NA	NA	185	44.6	5
Al-Asadi, 2015 (38)	Iraq	251	Cross-Sectional Study	NA/ICD-10	NA	NA	173	68.9	5
Oladeji, 2018 (33)	Nigeria	110	Cross-Sectional Study	NA/PHQ-9	NA	NA	58	52.7	5
Drosdzol, 2009 (41)	Polish	206	Cross-Sectional Study	BAI/BDI	32	15.53	73	35.44	6
Tuan M Vo, 2019 (31)	Vietnam	401	Cross-Sectional Study	NA/PHQ-9	NA	NA	49	12.2	5
Wu, 2014 (72)	China	288	Cross-Sectional Study	NA/CES-D10	NA	NA	65	22.6	3
Yusuf, 2016 (73)	Pakistan	100	Cross-Sectional Study	DASS 21/DASS 21	70	70	79	79	3
Sezgin, 2016 (37)	Turkey	100	Cross-Sectional Study	HADS/HADS	31	31	43	43	5
Cui, 2021 (30)	China	536	Cross-Sectional Study	HADS/HADS	150	27.9	226	42.2	6
Noël, 2022 (29)	China	42	Cross-Sectional Study	HADS/HADS	23	55	4	10	5
Yang, 2023 (23)	Thailand	150	Cross-Sectional Study	NA/OI-21	NA	NA	10	6.7	5
Niazi, 2024 (20)	Afghanistan	205	Cross-Sectional Study	NA/PHQ-9	NA	NA	162	79	5
Hasan, 2023 (26)	Bangladesh	300	Cross-Sectional Study	DASS 21/DASS 21	165	55	179	59.7	4
Makanjuola, 2010 (40)	Nigeria	160	Cross-Sectional Study	GHQ-30/GHQ-30	18	11.3	60	37.5	5
Zhang, 2022 (27)	China	1,247	Cross-Sectional Study	SAS/SDS	168	13.5	117	9.4	6
Beyene, 2025 (19)	Ethiopia	349	Cross-Sectional Study	NA/PHQ-9	NA	NA	147	42.1	6
Abdallah, 2024 (21)	Egyptian	236	Cross-Sectional Study	HAM-A/HAM-D	83	35.17	138	58.47	6
Leenakshi, 2023 (25)	Indian	112	Cross-Sectional Study	DASS 21/DASS 21	98	87.5	94	83.9	4
Ma, 2018 (74)	China	185	Case–Control Study	NA/SDS	NA	NA	87	47	7

NA, No data available.

### 3.3 Meta-analysis results

#### 3.3.1 Overall prevalence

**Figure 4:**
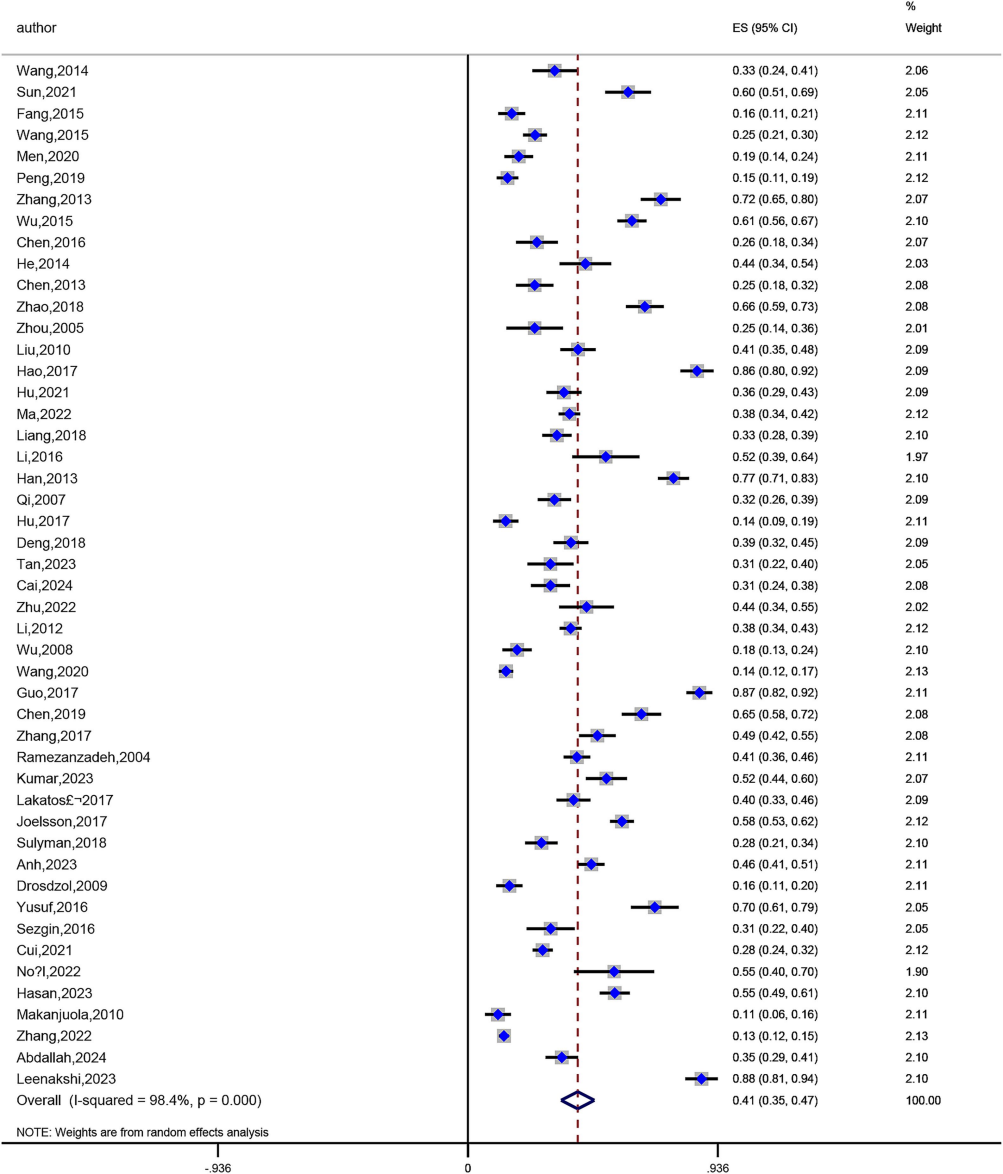
Meta-analysis of anxiety prevalence rates.

**Figure 5:**
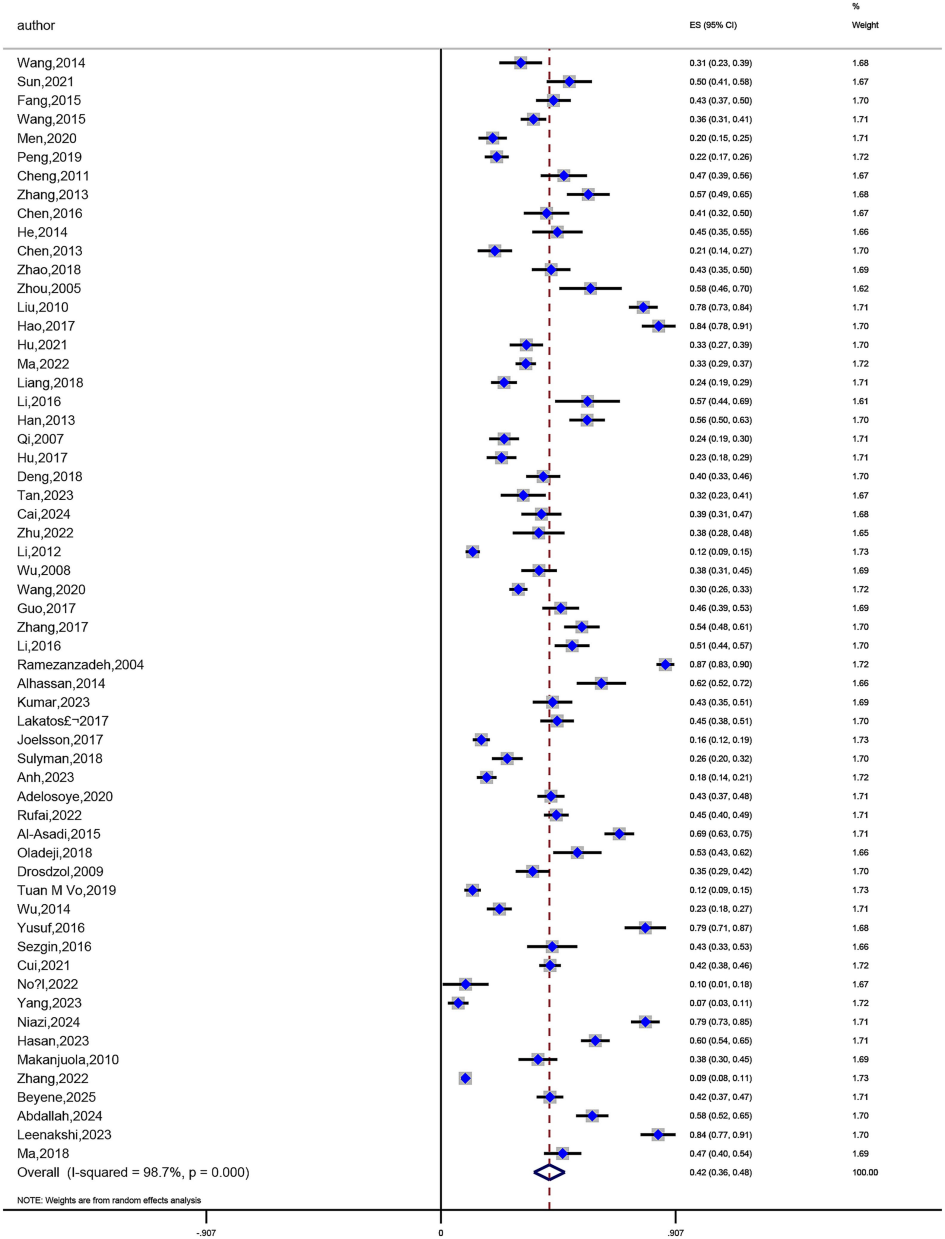
Meta-analysis of depression prevalence rates.

The prevalence of anxiety in infertile women was reported in 48 studies (14, 15, 17, 18, 21, 22, 24–27, 29, 30, 32, 34, 35, 37, 40–65, 67–71, 73) among the included studies. Prevalence rates varied greatly, ranging from a minimum of 11.3% (40) to a maximum of 87.5% (25). Significant study heterogeneity was found in a meta-analysis of these 48 publications (I^2^ = 98.4%, *p* < 0.01). According to a pooled analysis using a random-effects model, 41% of infertile women had anxiety, 95% CI (0.35, 0.47) (Figure 4). The prevalence of depression in infertile women was also reported in 59 studies (15–58, 60–74), with prevalence rates ranging from 9.4% (27) to 86.8% (69). Significant heterogeneity was also found in the meta-analysis of these 59 studies (I^2^ = 98.7%, *p* < 0.01). According to a random-effects model, 42% of infertile women had depression, 95% CI (0.36, 0.48) (Figure 5).

#### 3.3.2 Subgroup analysis

The subgroups were grouped according to their age (<25, 25–35, >35), regions (Asia, Europe, Africa), publication years (2004–2010, 2011–2015, 2016–2020, 2021–2025), and assessment tools (anxiety including SAS, HADS, DASS-21; depression including SDS, PHQ-9, HADS, DASS-21, BDI) because of the substantial heterogeneity among them. Consequently, a pooled analysis was conducted using a random-effects model.

##### 3.3.2.1 Anxiety subgroup analysis

**Figure 6:**
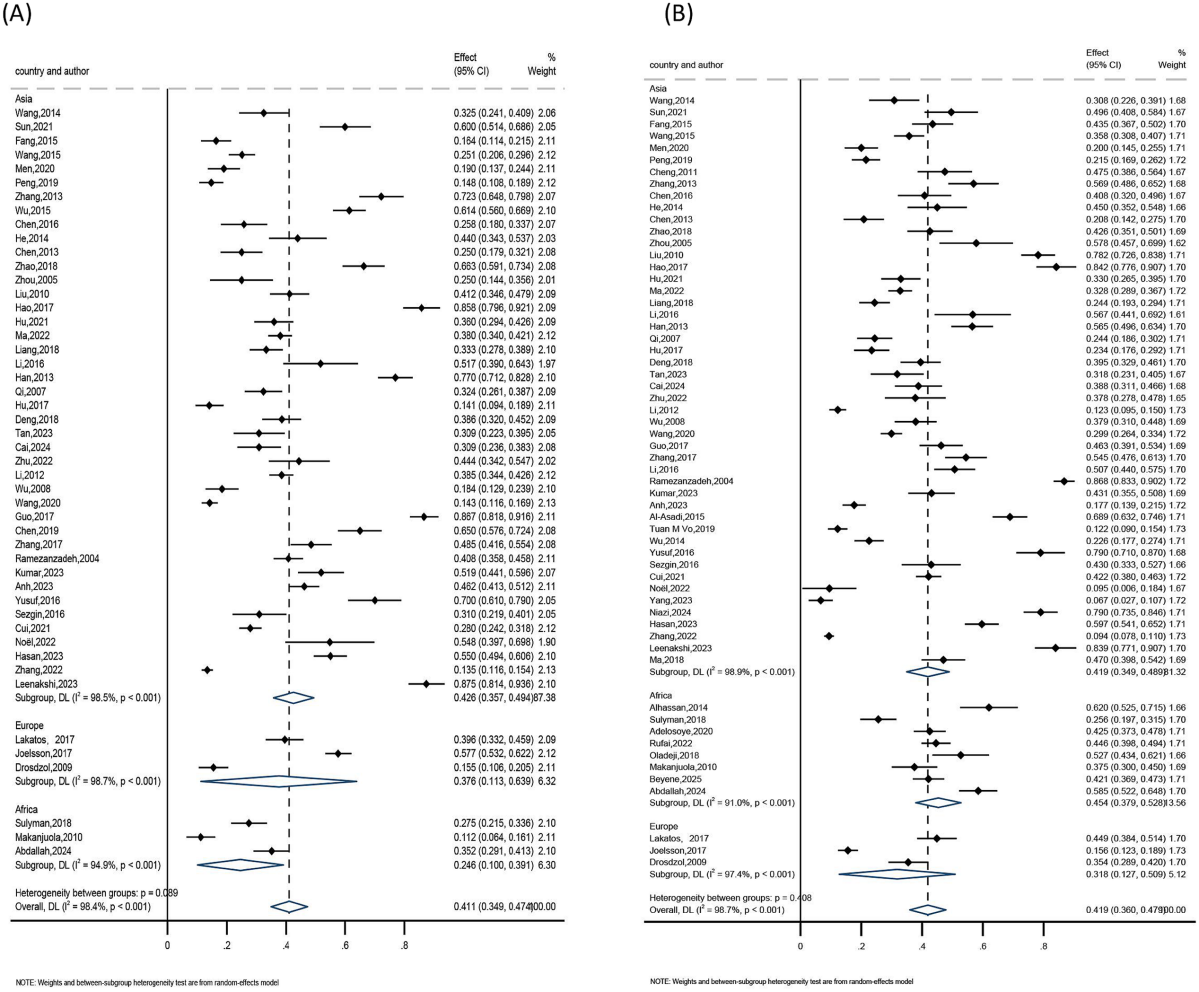
Subgroup analysis by regions: **(A)** anxiety and **(B)** depression.

**Figure 7:**
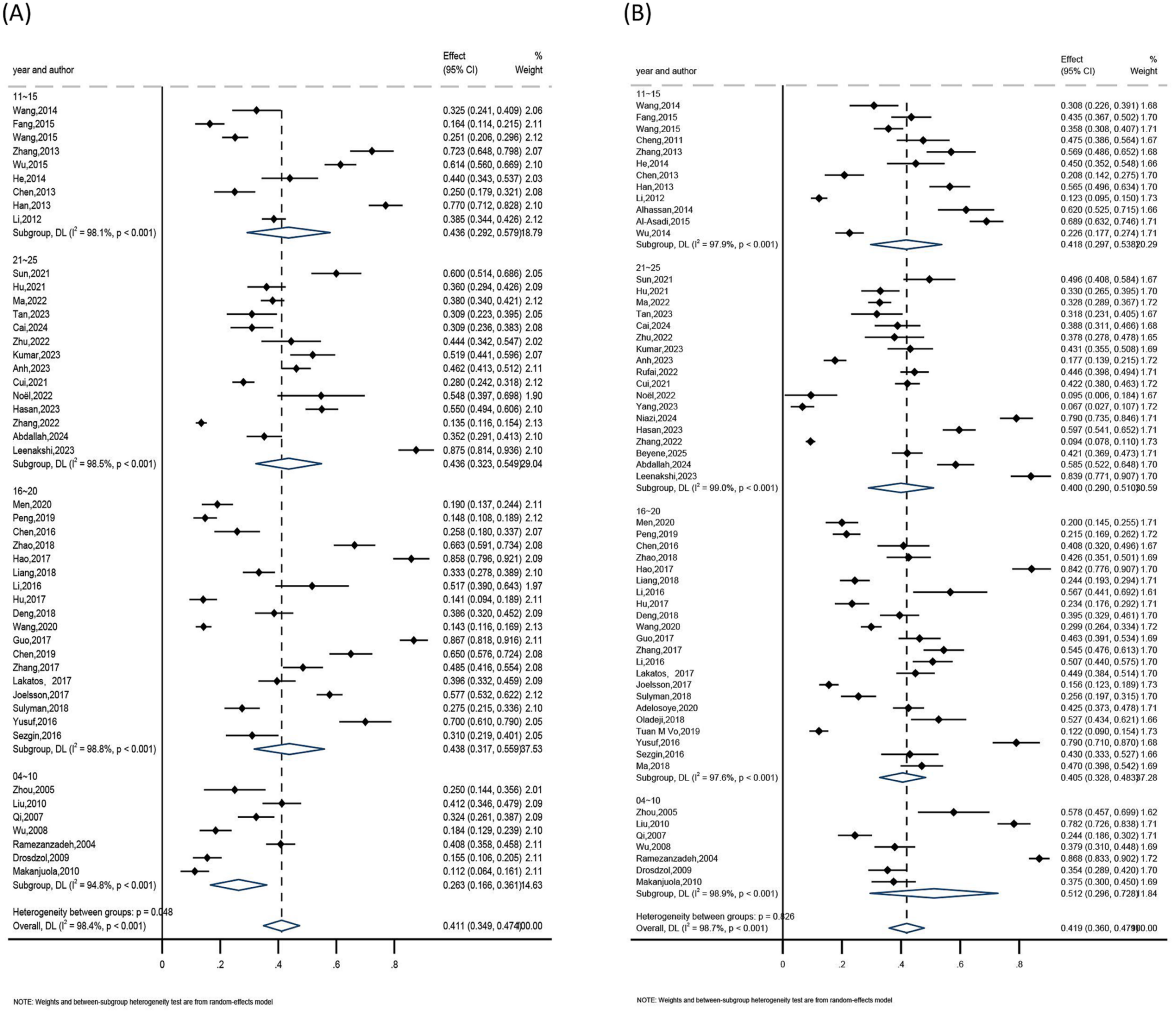
Subgroup analysis by publication year: **(A)** anxiety and **(B)** depression.

**Figure 8:**
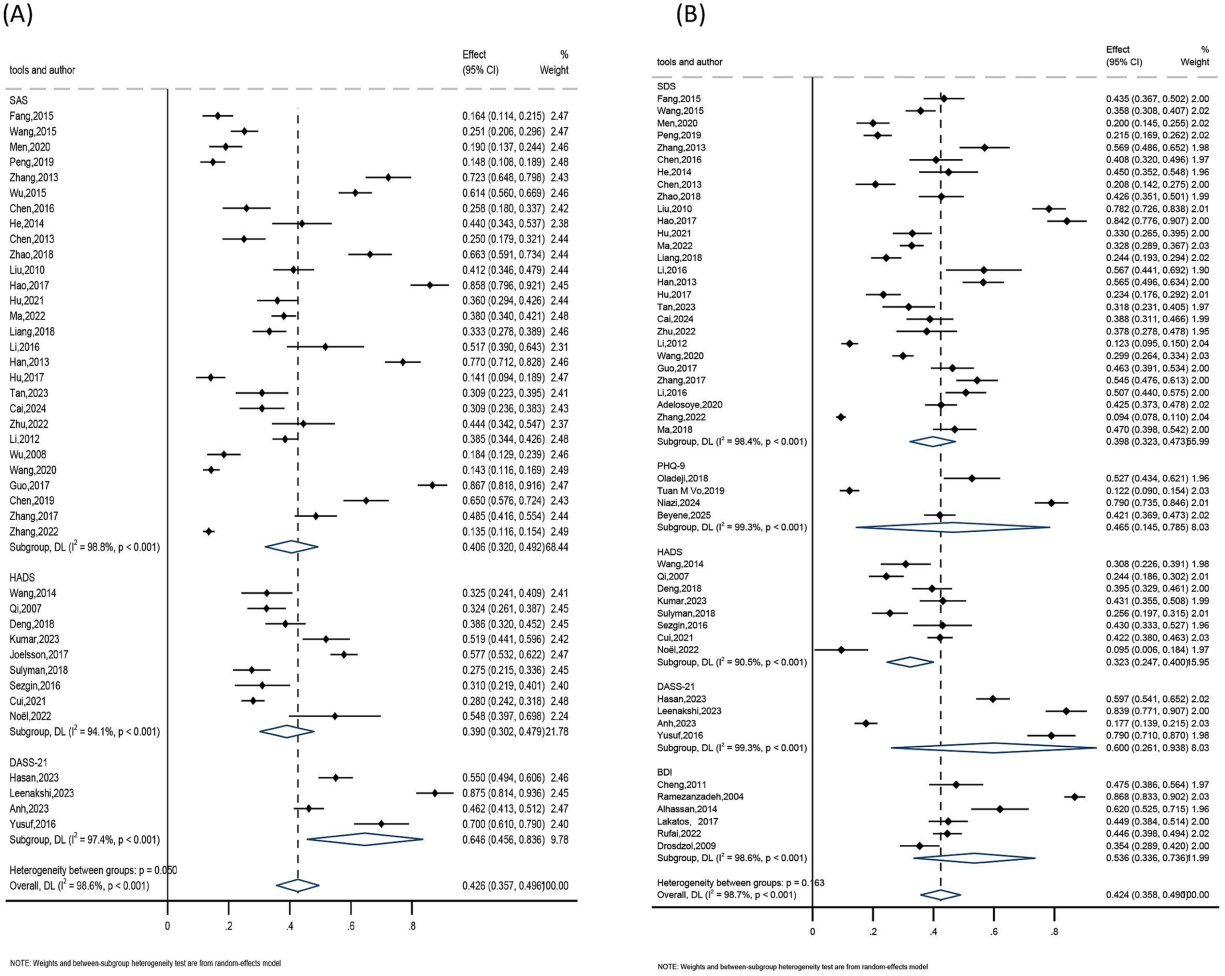
Subgroup analysis by assessment tools: **(A)** anxiety and **(B)** depression.

**Figure 9:**
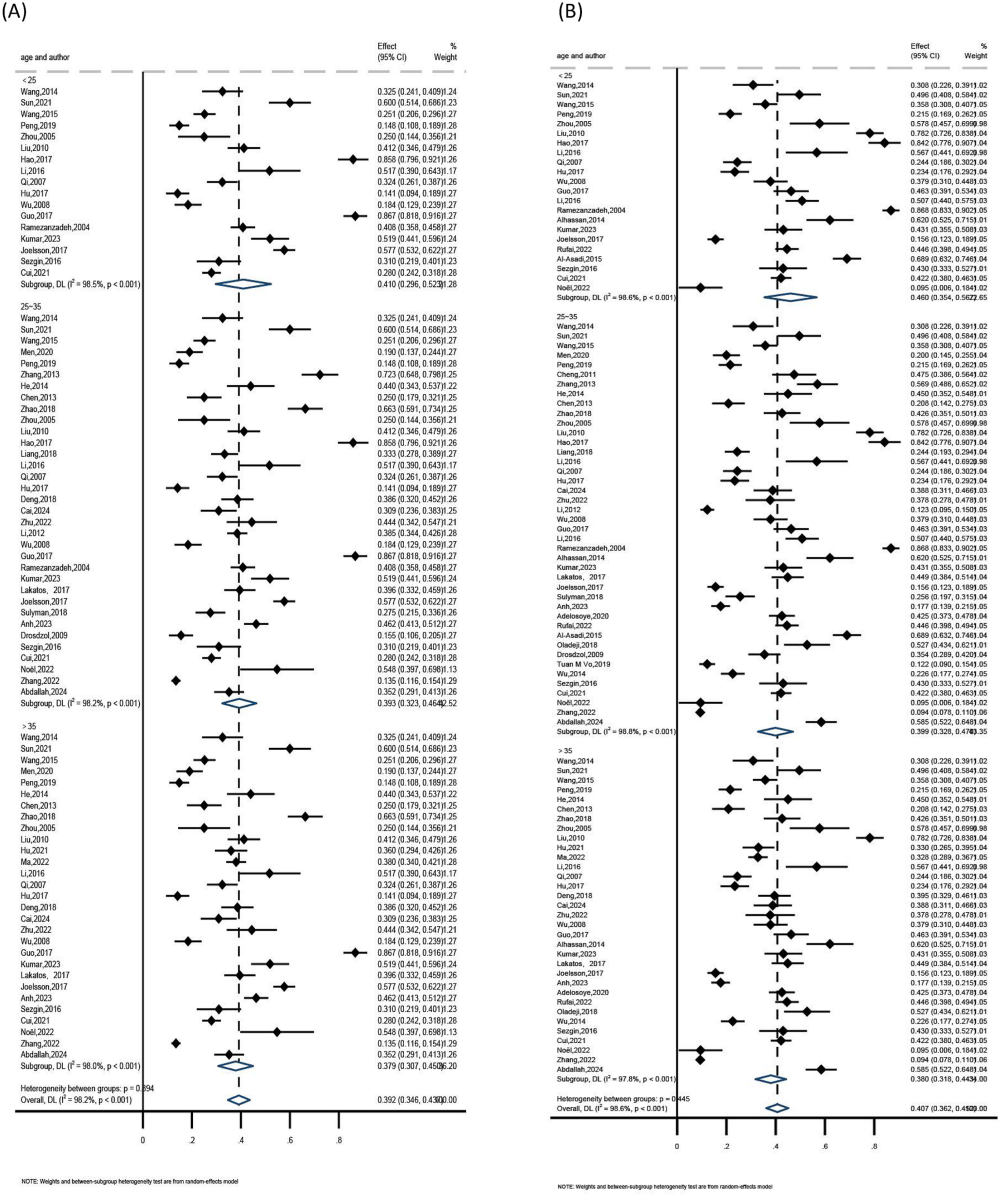
Subgroup analysis by age: **(A)** anxiety and **(B)** depression.

The findings showed that the highest anxiety detection rate was seen among female infertility patients under 25 years old, living in Asia, with publications from 2016 to 2020, and utilizing the DASS-21 assessment method. The age group under 25 had the highest detection rate (41%), followed by the 25–35 age group (39.3%), and the age group over 35 (37.9%). The greatest detection rate was 42.6% in Asia, compared to 37.6% in Europe and 24.6% in Africa. The detection rate was 43.8% for studies published between 2016 and 2020, 43.6% for those published between 2011 and 2015 and 2021 and 2025, and 26.3% for those published between 2004 and 2010. At 64.6%, the DASS-21 assessment tool had the highest detection rate, followed by SAS at 40.6%, while the HADS assessment tool had the lowest detection rate at 39.0%. Refer to Table 2 and Figures 6–9 for more information.

**Table 2:** Subgroup analysis of anxiety prevalence in infertile women: stratified by region, publication year, assessment tool, and age group.

Subgroup	Number of articles	Heterogeneity test		Effects model	Prevalence (95% CI)(%)	p-value
		I^2^ (%)	*p*-value			
Countries						<0.001
Asia	42 (14, 15, 17, 18, 22, 24–27, 29, 30, 37, 42–65, 67–71, 73)	98.5	<0.001	Random	42.6(35.7 ~ 49.4)	
Africa	3 (21, 32, 40)	94.9	<0.001	Random	24.6(10.0 ~ 39.1)	
Europe	3 (34, 35, 41)	98.7	<0.001	Random	37.6(11.3 ~ 63.9)	
Years						<0.001
04 ~ 10	7 (40, 41, 67–71)	94.8	<0.001	Random	26.3(16.6 ~ 36.1)	
11 ~ 15	9 (15, 17, 59–65)	98.1	<0.001	Random	43.6(29.2 ~ 57.9)	
16 ~ 20	18 (14, 15, 17, 32, 34, 35, 37, 47–65, 73)	98.8	<0.001	Random	43.8(31.7 ~ 55.9)	
21 ~ 25	14 (18, 21, 22, 24–27, 29, 30, 42–46)	98.5	<0.001	Random	43.6(32.3 ~ 54.9)	
Tools						<0.001
SAS	28 (14, 15, 27, 42–51, 53–65, 67, 70)	98.8	<0.001	Random	40.6(32.0 ~ 49.2)	
HADS	9 (17, 22, 29, 30, 32, 34, 37, 52, 71)	94.1	<0.001	Random	39.0(30.2 ~ 47.9)	
DASS-21	4 (24–26, 73)	97.4	<0.001	Random	64.6(45.6 ~ 83.6)	
Age						<0.001
<25	17 (17, 18, 22, 30, 34, 37, 49, 54–57, 60, 67–71)	98.5	<0.001	Random	41.0(29.6 ~ 52.3)	
25 ~ 35	34 (15, 17, 18, 21, 22, 24, 27, 29, 30, 32, 34, 35, 37, 41, 42, 44, 48–52, 54–57, 60, 62, 64, 65, 67–71)	98.2	<0.001	Random	39.3(32.3 ~ 46.4)	
>35	29 (17, 18, 21, 22, 24, 27, 29, 30, 34, 35, 37, 42, 44–46, 48–50, 52, 54, 56, 57, 60, 62, 64, 67, 68, 70, 71)	98	<0.001	Random	37.9(30.7 ~ 45.0)	

##### 3.3.2.2 Depression subgroup analysis

Results showed that the highest rate of depression identification was seen in female infertility patients under 25 years old, living in Africa, with publications from 2004 to 2010, and utilizing the DASS-21 assessment method. The age group under 25 had the highest detection rate (46.0%), followed by the 25–35 age group (39.9%), and the age group over 35 (38.0%). Africa had a 45.4% detection rate, compared to 41.9 and 31.8% in Asia and Europe, respectively. The detection rate was 51.2% for studies published between 2004 and 2010, 41.8% for those published between 2011 and 2015, and 40.5 and 40.0% for those published between 2016 and 2020 and 2021 and 2025, respectively. The greatest detection rate was 60.0% for the DASS-21 assessment tool, followed by 53.6% for the BDI, 46.5% for the PHQ-9, 39.8% for the SDS, and 32.3% for the HADS. Refer to Table 3 and Figures 6–9 for more information.

**Table 3:** Subgroup analysis of depression prevalence in infertile women: stratified by region, publication year, assessment tool, and age group.

Subgroup	Number of articles	Heterogeneity test		Effects model	Prevalence (95% CI)(%)	*p*-value
		I^2^ (%)	*p*-value			
Countries						<0.001
Asia	48 (15, 17, 18, 20, 22–27, 29–31, 36–38, 42–58, 60–74)	98.9	<0.001	Random	41.9 (34.9, 48.9)	
Africa	8 (16, 19, 21, 28, 32, 33, 39, 40)	91	<0.001	Random	45.4 (37.9, 52.8)	
Europe	3 (34, 35, 41)	97.4	<0.001	Random	31.8 (12.7, 50.9)	
Years						<0.001
04 ~ 10	7 (40, 41, 67–71)	98.9	<0.001	Random	51.2 (29.6, 72.8)	
11 ~ 15	12 (15, 17, 38, 39, 60–66, 72)	97.9	<0.001	Random	41.8 (29.7, 53.8)	
16 ~ 20	22 (16, 31–37, 47–58, 73, 74)	97.6	<0.001	Random	40.5 (32.8, 48.3)	
21 ~ 25	18 (18–30, 42–46)	99	<0.001	Random	40.0 (29.0, 51.0)	
Tools						<0.001
SDS	28 (15, 16, 27, 36, 42–51, 53–58, 60–65, 70, 74)	98.4	<0.001	Random	39.8 (32.3, 47.3)	
HADS	8 (17, 22, 29, 30, 32, 37, 52, 71)	90.5	<0.001	Random	32.3 (24.7, 40.0)	
BDI	6 (28, 35, 39, 41, 66, 69)	98.6	<0.001	Random	53.6 (33.6, 73.6)	
DASS-21	4 (24–26, 73)	99.3	<0.001	Random	60.0 (26.1, 93.8)	
PHQ-9	4 (19, 20, 31, 33)	99.3	<0.001	Random	46.5 (14.5, 78.5)	
Age						<0.001
<25	22 (17, 18, 22, 28–30, 34, 36–39, 49, 54–57, 60, 67–71)	98.6	<0.001	Random	46.0(35.4 ~ 56.7)	
25 ~ 35	42 (15–18, 21, 22, 24, 27–39, 41, 42, 44, 48–51, 54–57, 60, 62, 64–72)	98.8	<0.001	Random	39.9(32.8 ~ 47.0)	
>35	33 (16–18, 21, 22, 24, 27–30, 33–35, 37, 39, 42, 44–46, 49, 50, 52, 54, 56, 57, 62, 64, 67, 68, 70–72)	97.8	<0.001	Random	38.0(31.8 ~ 44.3)	

### 3.4 Meta-analysis of risk factors

**Figure 10:**
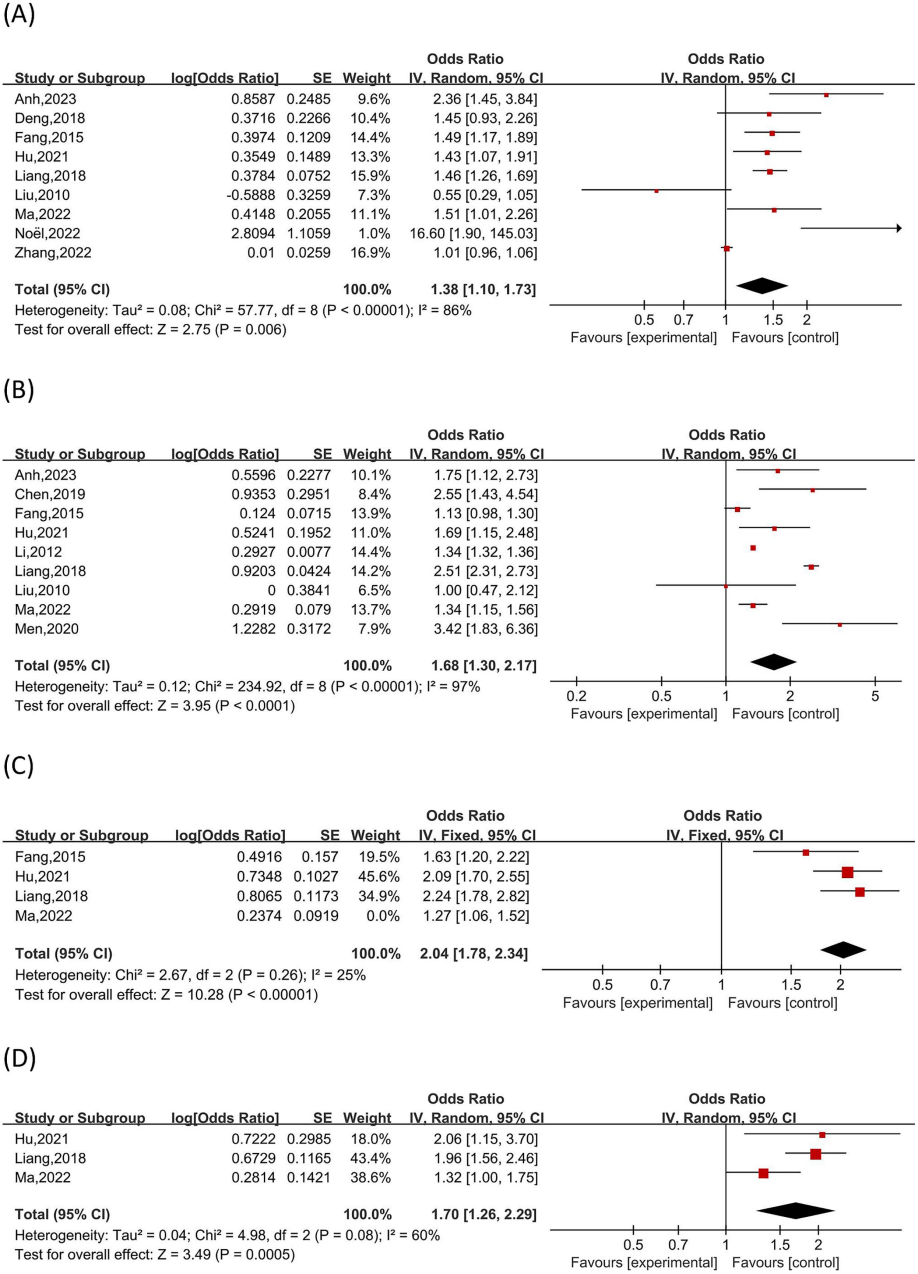
Risk factors for anxiety. **(A)**: age, **(B)**: duration of infertility, **(C)**: treatment expenses, **(D)**: Lack of knowledge about ART-related Information.

**Figure 11:**
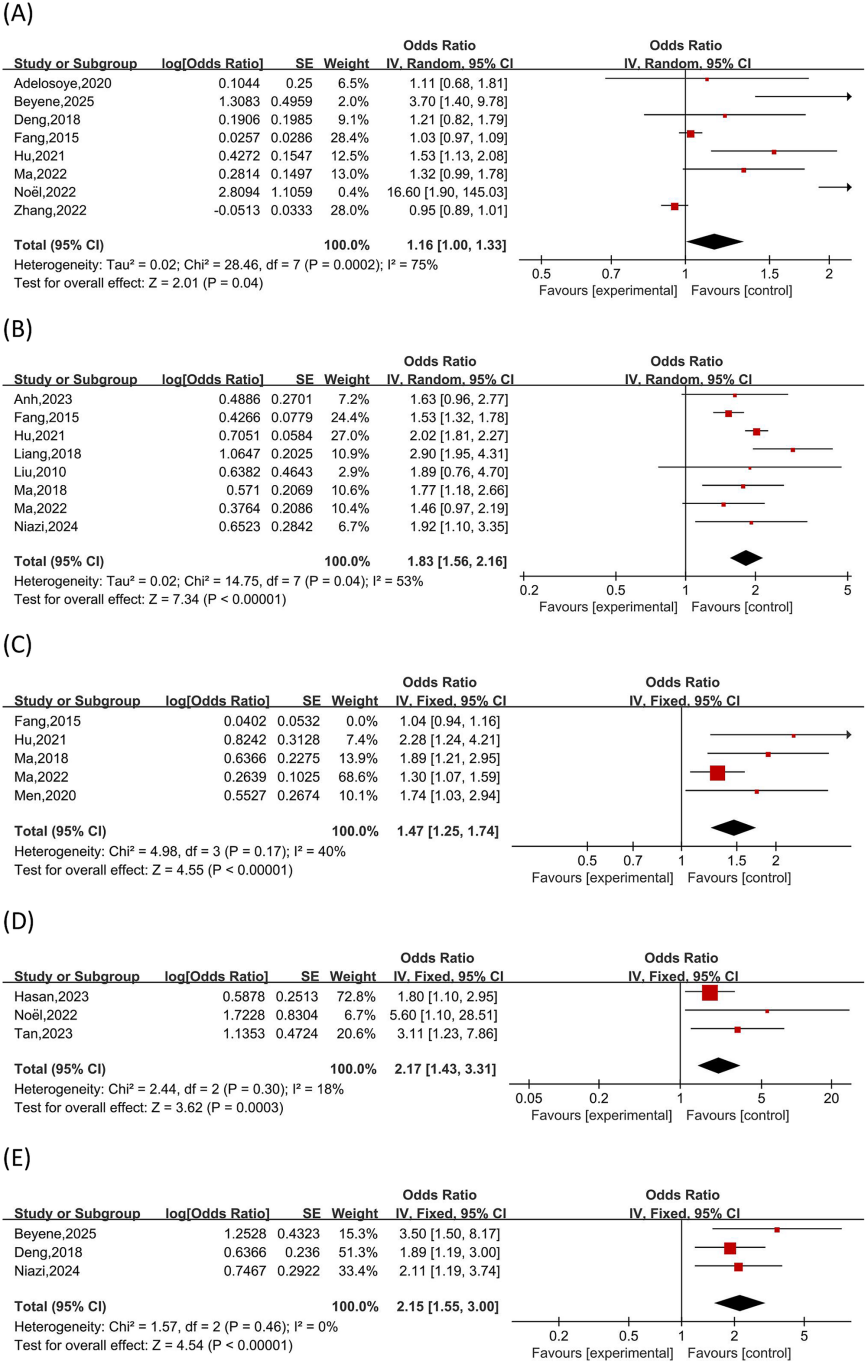
Risk factors for depression. **(A)**: age. **(B)**: duration of infertility. **(C)**: treatment costs. **(D)**: history of miscarriage. **(E)**: primary infertility.

Of the 61 publications in this investigation, 25 discussed the contributing variables to female infertility patients’ anxiety (n = 17) or depression (n = 23). Thirty-four influencing factors for depression and twenty-five influencing factors for anxiety were retrieved. Seven influencing factors linked to anxiety and eight relating to depression were identified by a meta-analysis of influencing factors with ≥3 studies. Six of these factors—age, residence, family income, absence of reproductive history, length of infertility, and treatment expenses—were shared by both anxiety and depression. Refer to (Figures 10, 11) for more information.

#### 3.4.1 Patient factors

##### 3.4.1.1 Age

The association between age and anxiety in female infertility patients was investigated in nine studies (24, 27, 29, 45, 46, 51, 52, 61, 70). There was notable variation among the studies (I^2^ = 86%, *p* < 0.01). A random-effects model demonstrated age as a correlate of anxiety in female infertility patients [OR = 1.38, 95% CI (1.10, 1.73), p < 0.01]. Nine studies (16, 27, 29, 39, 45, 46, 52, 61, 70) examining age and depression showed significant heterogeneity (I^2^ = 75%, p < 0.01), with pooled results confirming age as a significant risk factor for depression [OR = 1.16, 95% CI (1.00, 1.33), *p* < 0.05].

##### 3.4.1.2 Place of residence

Three studies (48, 61, 65) examining residence and anxiety in infertile women showed significant heterogeneity (I^2^ = 86%), with random-effects models indicating no significant association [OR = 1.20, 95%CI(0.45, 3.19)]. Three depression studies (19, 61, 67) demonstrated heterogeneity (I^2^ = 57%) but consistently showed no significant risk association with residence [OR = 0.77, 95%CI(0.36, 1.63)].

##### 3.4.1.3 Family income

Family income was found to have a significant heterogeneous effect on anxiety (24, 45, 46, 51, 52, 70) and depression (24, 28, 45, 46, 52, 70) in female infertility patients in six studies (anxiety I^2^ = 89%, *p* < 0.01; depression I^2^ = 71%, p < 0.01). Family income is not a significant risk factor for either anxiety [OR = 1.25, 95% CI (0.92, 1.70), *p* > 0.01] or depression [OR = 1.12, 95% CI (0.85, 1.47), p > 0.01], according to the results of a random-effects model used for pooled analysis.

##### 3.4.1.4 No reproductive history

The association between anxiety and a lack of reproductive history in female infertility patients was investigated in four studies (27, 45, 46, 52). There was notable variation among the studies (I^2^ = 90%, *p* < 0.01). Using a random-effects model for pooled analysis, the results showed [OR = 1.47, 95% CI (0.73, 2.97), *p* > 0.01]. However, this pooled estimate must be interpreted with extreme caution due to substantial heterogeneity. The wide confidence interval and high I^2^ value indicate that the true effect may vary considerably across different populations or study settings, and no consistent association can be confirmed. Three studies (45, 46, 52) examined the association between depression and a lack of reproductive history in female infertility patients, with heterogeneity among studies (I^2^ = 71%, *p* = 0.03). The results were obtained using a random-effects model, revealing [OR = 1.29, 95% CI (0.78, 2.13), *p* > 0.01]. According to both findings, female infertility patients’ lack of reproductive history is not a major risk factor for either anxiety or depression.

##### 3.4.1.5 History of miscarriage

The history of Miscarriage and depression in female infertility patients was the subject of three studies (26, 29, 43), with acceptable heterogeneity between the studies (I^2^ = 18%, p > 0.01). According to the results of a fixed-effects model for pooled analysis, treatment expenditures are a significant risk factor for depression in female infertility patients [OR = 2.17, 95% CI (1.43, 3.31), *p* < 0.01].

#### 3.4.2 Disease factors

##### 3.4.2.1 Duration of infertility

Nine studies (14, 24, 45, 46, 48, 51, 61, 65, 70) assessed the relationship between infertility length and anxiety in infertile women. Given the considerable heterogeneity (I^2^ = 97%, p < 0.01), a random-effects model was used, and the result should be interpreted with caution. The resulting pooled estimate of [OR = 1.68, 95% CI (1.30, 2.17, p < 0.01)] suggests that a longer period of infertility is correlated with a higher prevalence of anxiety. The length of infertility and depression were the subjects of eight investigations (20, 24, 45, 46, 51, 61, 70, 74). The study by Fangzhou et al. (61) was found to be the primary source, and moderate heterogeneity was noted (I^2^ = 53%, *p* > 0.01). Heterogeneity disappeared after this sample was excluded, and a fixed-effects model yielded a pooled [OR = 1.83, 95% CI (1.56, 2.16, *p* < 0.01)], suggesting that higher infertility duration is associated with a higher risk of depression in female infertility patients.

##### 3.4.2.2 Primary infertility

There was no variation in the findings of three studies (19, 20, 52) that looked at the connection between depression and primary infertility in female infertility patients (I^2^ = 0%, *p* > 0.01). Primary infertility is a substantial risk factor for depression in female infertility patients, according to the results, which were analyzed using a fixed-effects model [OR = 2.15, 95% CI (1.55, 3.00), *p* < 0.01].

#### 3.4.3 Treatment factors

##### 3.4.3.1 Lack of knowledge about ART-related information

Three studies (45, 46, 51) looked at the relationship between anxiety and ignorance of ART-related information in female infertility patients. Because of the moderate heterogeneity (I^2^ = 60.0%, *p* > 0.01), a random-effects model was used. The pooled estimate was [OR = 1.70, 95% CI (1.26, 2.29), *p* < 0.01], suggesting that a substantial risk factor for anxiety is a lack of understanding about ART.

#### 3.4.4 Economic factors

##### 3.4.4.1 Treatment costs

The relationship between treatment expenses and anxiety in infertile women was assessed in four studies (45, 46, 51, 61). There was significant heterogeneity observed (I^2^ = 85%, *p* < 0.01), which was related to the Ma Li et al. study (45). Heterogeneity was lowered to a non-significant level (I^2^ = 25%, *p* > 0.01), following the exclusion of this study. A pooled OR of 2.04 (95% CI: 1.78–2.34; *p* < 0.01) was subsequently obtained using a fixed-effects model, suggesting that greater treatment expenses constitute a significant risk factor for anxiety. The association between treatment expenses and depression in female infertility patients was evaluated in five studies (45, 46, 48, 61, 74). Significant heterogeneity was noted (I^2^ = 76%, *p* < 0.01), and the study by Fangzhou et al. (61) was determined to be the principal source by sensitivity analysis. Higher treatment expenses are a significant risk factor for depression, as evidenced by the pooled OR of 1.47 (95% CI: 1.25–1.74; p < 0.01) obtained from a fixed-effects model when homogeneity was attained (Figures 2, 3).

### 3.5 Sensitivity analysis

**Figure 12:**
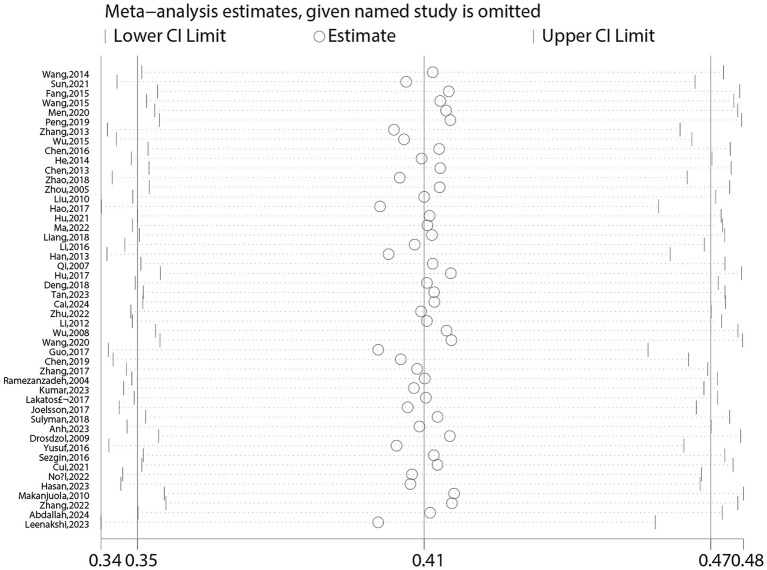
Sensitivity analysis for pooled prevalence of anxiety in infertile women.

**Figure 13:**
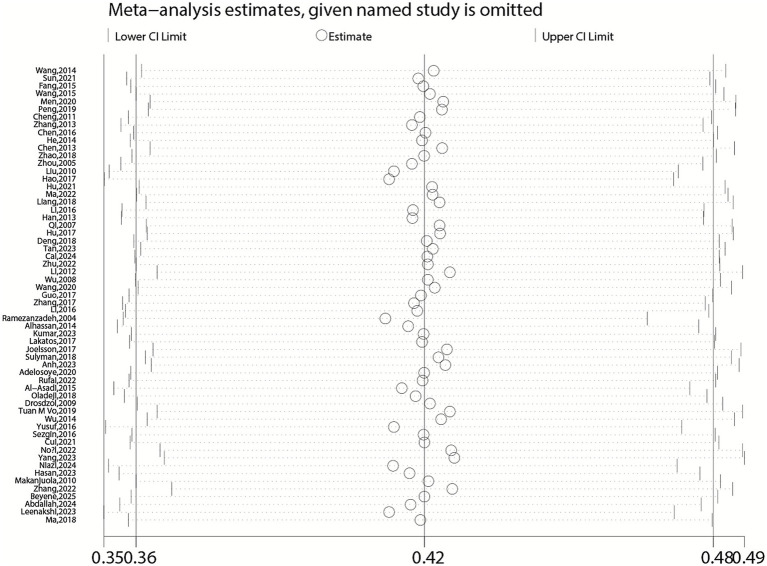
Sensitivity analysis of depression.

Sensitivity analysis was conducted by successively excluding each study to assess its impact on the overall pooled estimate (Figures 12, 13). Additionally, we performed a quantitative influence analysis using the “metainf” command in Stata, which excluded one study at a time and calculated the pooled estimate. The results indicated that the exclusion of any single study had no significant impact on the overall positive rate of anxiety and depression in female infertility patients, suggesting that the results of the meta-analysis were relatively stable (Figures 4–11).

## 4 Discussion

### 4.1 High prevalence of anxiety or depression in female infertility patients

This systematic review and meta-analysis comprehensively integrates recent Chinese and English literature to investigate the prevalence of comorbid depression and anxiety in female infertility patients, as well as the risk factors that influence these conditions. While previous meta-analyses (8, 9) have included studies in both languages, our study provides an updated synthesis with a broader scope of risk factors and more recent evidence. According to the meta-analysis’s findings, 41% of female infertility patients had anxiety, 95% CI (0.35, 0.47), and 42% had depression, 95% CI (0.36, 0.48), both of which are comparatively high rates. These results are greater than the reported prevalences of anxiety (20%) and depression (26%), respectively, in male infertility patients (75), and they are in line with those reported by Kiani et al. (9). According to research, women face more social pressure than men do, and giving up the strong desire to have children causes them to suffer more psychologically—a cost that men do not usually bear (76, 77). Age, the length of infertility, and the expense of treatment are frequent risk factors for anxiety and depression in infertile women, according to this pooled review. Moreover, primary infertility and a history of miscarriage are risk factors exclusive to depression, but ignorance of ART-related information is a correlate of anxiety.

Subgroup analysis revealed distinct geographical patterns in the prevalence of anxiety and depression among infertile women. The pooled prevalence of anxiety was highest in Asia, followed by Europe, and lowest in Africa. In contrast, the pooled prevalence of depression was highest in Africa, followed by Asia, and was lowest in Europe. The distinct regional patterns may be attributed to differing dominant stressors; the high anxiety prevalence in Asia could be primarily driven by socio-cultural pressures, such as intense stigma and the imperative to fulfill familial expectations (9, 77). In contrast, the high depression prevalence in Africa may be more closely linked to socioeconomic barriers, including catastrophic treatment costs and limited access to fertility care, which can foster feelings of hopelessness (78, 79). In addition, it may also be related to health and medical services. The majority of high-, middle-, and low-income nations are found in Europe, Asia, and Africa, respectively, according to the World Bank’s classification of nations. According to earlier research, the proportion of health spending to GDP in low-income nations is much lower than in high-income nations; it ranges from 3.4% in Nigeria to 13.9% in the United States (80). Prevalence rates varied across studies conducted in different years, but no consistent temporal pattern was observed. Growing awareness of mental health issues, the clinical acceptance of standardized psychological disorder screening procedures, and the increased psychological stress that infertility patients suffer over time could all be contributing factors to this increase. The prevalence rates also varied according to the assessment instruments that were employed, most likely as a result of variations in the instruments’ content, applicability, and assessment cut-off values. In particular, the HADS-measured prevalence rates of depression and anxiety were comparatively lower than those of other instruments. While DASS-21 is appropriate for a variety of demographics, HADS is intended for non-psychiatric patients and is useful in differentiating symptoms of anxiety, depression, and other physical problems. Additionally, SAS and SDS have drawbacks because they depend on the participants’ self-reports of how severe their symptoms are, which puts extra pressure on the assessing doctors. It’s interesting to note that, contrary to prior research (81) that suggested anxiety and depression inclinations rise with age, the prevalence rates of anxiety and depression comorbidity among infertile women did not significantly change among age groups. This disparity may be explained by variations in the definitions and inclusion criteria used in different studies. Some studies, for example, exclusively looked at older women (>35 years) who were infertile, while the majority included women of reproductive age.

### 4.2 Influencing factors of anxiety or depression in female infertility patients

#### 4.2.1 Patient factors

In line with the findings of Chiaffarino et al. (82), this study discovered that age is independently associated with anxiety and depression in female infertility patients. This may be due to the fact that women’s fertility naturally decreases with age. Reduced antioxidant capacity, a lowered energy supply, and an increased rate of oocyte loss due to apoptosis all contribute to lower oocyte quality as people age (83), which in turn affects conception rates. Furthermore, as mothers age, their chance of having birth abnormalities may also increase (84). According to research, infertile women over 35 who have urgent fertility desires are more likely to feel depressed (85). Long-term infertility also correlated with a great deal of psychological suffering, with the ensuing symptoms of worry and depression getting worse over time (86).

Kolte et al. (87) conducted a survey of 301 recurrent miscarriage patients and found that the incidence of moderate to severe depression was five times higher than that observed in general women of reproductive age. Another study (88) compared the occurrence of anxiety and depression among patients with recurrent miscarriage, single miscarriage, and no miscarriage history, finding that both recurrent and single miscarriage patients had significantly elevated SDS and SAS scores. This suggests that a history of miscarriage has adverse psychological impacts on patients. The results of this study align with these findings, showing that patients with a miscarriage history suffer not only physical harm, such as cervical incompetence and endometrial damage, which contribute to conception difficulties, but also face an increased likelihood of recurrent miscarriages. This condition can create pregnancy-related fear and heightened psychological stress, leading to negative emotional outcomes such as anxiety and depression; Studies have shown that anxiety and depression in patients with previous miscarriages may persist into subsequent pregnancies and into nearly 3 years postpartum (89).

#### 4.2.2 Disease factors

Research shows different findings regarding the duration of infertility and the severity of depression. While Gourounti et al. (90) reported no significant change in depression scores with prolonged duration, our study identified an association between duration of infertility and anxiety-depression comorbidities. Wang Xiaohong et al. (91) demonstrated that anxiety and depressive symptoms worsened with infertility duration below 6 years, but no significant correlation over 6 years, using SAS/SDS in 320 patients. Chronic infertility with repeated treatment failures can lead to self-deprecation, guilt, and decreased confidence in fertility. One’s chances of getting pregnant may be adversely affected by these variables, which may contribute to a vicious cycle that is associated with long-term psychiatric illnesses. Additionally, individuals who have had infertility for a longer duration may have increased financial stresses, as well as social and familial pressures, which can worsen depressive and anxious symptoms and contribute to mental health conditions like anxiety and depression (92).

Consistent with our findings, an Iranian study (93) showed that patients with primary infertility had significantly higher anxiety and depression scores than those with secondary infertility, indicating a higher sensitivity to depressive symptoms. This disparity stems from the more ambiguous treatment of those who have not had a previous successful pregnancy, combined with different social stigma—people with primary infertility often face derogatory labels such as “sexual impotence” or “genetic defects,” while people with secondary infertility retain evidence of prior fertility. Further supporting this, Gupta et al. (94) determined a significant correlation between social stigma and increased perceived stress levels and depression severity in patients undergoing ART.

#### 4.2.3 Treatment factors

This study found that limited awareness of assisted reproductive technologies may increase the risk of anxiety in infertile women. While antiretroviral therapy has gained attention as an effective treatment, its long cycle, high cost, invasive procedures, and uncertainty about outcomes (95) can easily trigger negative emotions, including anxiety and hopelessness. Women on antiretroviral therapy endure repeated blood draws, vaginal examinations, ovulation induction injections, and egg retrieval procedures, which directly affect the outcome of conception and cause significant psychological distress (96). The Chinese Society of Reproductive Medicine reported that the clinical pregnancy rate of fresh embryo transfer cycles in China in 2019 was 51.95%, the live birth rate was 42.39%, and the multiple pregnancy rate was 26.04% (97). Multiple pregnancies greatly increase the risk of preterm birth and pose a serious threat to maternal and fetal health, with the 2019 ESHRE report documenting an extremely preterm birth rate of 12.2% for triplets compared to 3.3% for twins (98), suggesting that antiretroviral therapy carries significant risks while treating hope. As a result, infertility patients who are knowledgeable about ART are more likely to take a logical and impartial stance toward the treatment’s tenets, risks, and success rates, which promotes a more upbeat outlook and increased treatment compliance. Additionally, typical infertility drugs like gonadotropins, leuprorelin, and clomiphene have been linked to psychological problems like anxiety, melancholy, and irritability (99). Furthermore, studies have indicated that using Diane-35 may raise the chance of experiencing depressive symptoms (100).

#### 4.2.4 Economic factors

Treatment expenditures show a strong association with the prevalence of anxiety and depression in female infertility patients, according to the study’s findings. The direct cost of a single ART treatment cycle is two to three times greater than the yearly gross domestic product per capita in places such as Africa and Southeast Asia, despite the fact that assisted reproductive technology (ART) is a successful treatment for infertility (89, 101) Furthermore, several cycles are frequently needed to produce a successful pregnancy because the clinical pregnancy rate for assisted reproduction is only about 50% (97). Medical expenses are categorized as catastrophic health expenditures when they surpass 40% of household consumption (102). According to one study, 22% of South African individuals on ART had catastrophic medical expenses (78). Along with the direct costs of ART, patients also have to pay for various associated expenditures, such as lodging, transportation, meals during doctor’s appointments, and lost productivity from reduced job capability or absenteeism. Consequently, infertile women are subject to additional financial difficulties during the course of treatment in addition to the direct expenditures of medical therapies. These financial stresses are associated with severe psychological distress and an increased prevalence of depression and anxiety.

### 4.3 Clinical implications and future directions

These findings highlight the need to integrate psychosocial support into standard infertility care through the implementation of routine mental health screenings and the provision of accessible interventions, such as psychoeducation to bridge knowledge gaps and counseling to reduce financial strain, with future efforts focused on developing targeted interventions to mitigate risks and ultimately enhance overall care for affected women.

## 5 Limitations

This meta-analysis has several limitations, with significant heterogeneity between the included studies, which may be attributed to population characteristics, study design, statistical methods, diagnostic methods for anxiety and depression, and geocultural differences. Subgroup analysis was unable to pinpoint the cause of this heterogeneity, most likely as a result of the small number of included studies. Second, bias may be introduced because most of the included studies were cross-sectional, had insufficient data, and had ambiguous confounding variables. Third, some risk factors were pooled from a limited number of studies (as few as three), which may affect the precision of the pooled odds ratios. These findings should therefore be interpreted as preliminary and warrant validation by future research. Furthermore, this study only examined risk factors; protective factors were not examined, which restricts how thorough the results can be. To give more solid evidence for the next meta-analyses and systematic reviews, further research is therefore required.

## 6 Conclusion

In summary, this study’s findings show that anxiety and melancholy are quite prevalent among female infertility patients, and that these conditions are influenced by a variety of circumstances. Consequently, infertile women’s mental health concerns need to receive more attention. This meta-analysis identified several significant risk factors. Specifically, age, duration of infertility, and the treatment costs are common risk factors for both anxiety and depression in infertile women, according to this pooled review. Moreover, primary infertility and a history of miscarriage are risk factors exclusive to depression, whereas lack of ART-related information is a risk factor specific to anxiety. It is important to note that while other factors, such as place of residence, no reproductive history, and family income, were also investigated in our meta-analysis, they were not found to be statistically significant predictors for either anxiety or depression in this patient population. Stronger proof, however, requires more large-scale, high-quality investigations. Future studies should concentrate more on comprehending negative emotions, especially anxiety and depression, in female infertility patients.

## Data Availability

The original contributions presented in the study are included in the article/Supplementary material, further inquiries can be directed to the corresponding author.
