# Correction: Prevalence and risk factors of negative emotions in infertile women: a systematic review and meta-analysis

**DOI:** 10.3389/fpubh.2025.1760877

**Published:** 2026-01-16

**Authors:** Frontiers Production Office

**Affiliations:** Frontiers Media SA, Lausanne, Switzerland

**Keywords:** female infertility, anxiety, depression, related factors, prevalence, systematic review, meta-analysis

The reviewer Arbind Kumar was erroneously omitted as a reviewer.

The original version of this article has been updated.

