# Supplementary material for: Prevalence and risk factors of negative emotions in infertile women: a systematic review and meta-analysis
Source: Front Public Health. 2025 Dec 4;13:1701381. doi: 10.3389/fpubh.2025.1701381 (PMC12711472; doi:10.3389/fpubh.2025.1701381)

Supplementary Material

# Supplementary Tables

# Supplementary Table 1: Database search

# A combined subject and free word search was conducted using the following Chinese and English databases: PubMed, Web of Science, Embase, Cochrane Library, Sinomed, China National Knowledge Infrastructure (CNKI), VIP Database, and WanFang Database. Taking PubMed as an example:

| **PubMed** (from the inception to February 28, 2025), n=562 | |
| --- | --- |
| #1 | "Infertility"[Mesh] |
| #2 | (((((Infertility[Title/Abstract]) OR (Sterility, Reproductive[Title/Abstract])) OR (Reproductive Sterility[Title/Abstract])) OR (Sterility[Title/Abstract])) OR (Subfertility[Title/Abstract])) OR (Sub-Fertility[Title/Abstract]) |
| #3 | #1 OR #2 |
| #4 | "Anxiety"[Mesh] |
| #5 | ((((((((anxiety[Title/Abstract]) OR (Angst[Title/Abstract])) OR (Nervousness[Title/Abstract])) OR (Hypervigilance[Title/Abstract])) OR (Social Anxiety[Title/Abstract])) OR (Anxieties, Social[Title/Abstract])) OR (Anxiety, Social[Title/Abstract])) OR (Social Anxieties[Title/Abstract])) OR (Anxiousness[Title/Abstract]) |
| #6 | #4 OR #5 |
| #7 | "Depression"[Mesh] |
| #8 | (((((Depression[Title/Abstract]) OR (Depressive Symptoms[Title/Abstract])) OR (Depressive Symptom[Title/Abstract])) OR (Symptom, Depressive[Title/Abstract])) OR (Emotional Depression[Title/Abstract])) OR (Depression, Emotional[Title/Abstract]) |
| #9 | #7 OR #8 |
| #10 | "Mental Disorders"[Mesh] |
| #11 | (((((((((((((((((mental disorders[Title/Abstract]) OR (Mental Disorder[Title/Abstract])) OR (Psychiatric Disorders[Title/Abstract])) OR (Psychiatric Disorder[Title/Abstract])) OR (Psychiatric Diseases[Title/Abstract])) OR (Psychiatric Disease[Title/Abstract])) OR (Psychiatric Illness[Title/Abstract])) OR (Psychiatric Illnesses[Title/Abstract])) OR (Mental Illness[Title/Abstract])) OR (Illness, Mental[Title/Abstract])) OR (Mental Illnesses[Title/Abstract])) OR (Behavior Disorders[Title/Abstract])) OR (Diagnosis, Psychiatric[Title/Abstract])) OR (Psychiatric Diagnosis[Title/Abstract])) OR (Mental Disorders, Severe[Title/Abstract])) OR (Mental Disorder, Severe[Title/Abstract])) OR (Severe Mental Disorder[Title/Abstract])) OR (Severe Mental Disorders[Title/Abstract]) |
| #12 | #10 OR #11 |
| #13 | #6 OR #9 OR #12 |
| #14 | "Prevalence"[Mesh] |
| #15 | (((((((Prevalence[Title/Abstract]) OR (Prevalences[Title/Abstract])) OR (Point Prevalence[Title/Abstract])) OR (Point Prevalences[Title/Abstract])) OR (Prevalence, Point[Title/Abstract])) OR (Period Prevalence[Title/Abstract])) OR (Period Prevalences[Title/Abstract])) OR (Prevalence, Period[Title/Abstract]) |
| #16 | #14 OR #15 |
| #17 | "Epidemiology"[Mesh] |
| #18 | ((((Epidemiology[Title/Abstract]) OR (Social Epidemiology[Title/Abstract])) OR (Epidemiologies, Social[Title/Abstract])) OR (Epidemiology, Social[Title/Abstract])) OR (Social Epidemiologies[Title/Abstract]) |
| #19 | #17 OR #18 |
| #20 | #16 OR #19 |
| #21 | #3 AND #13 AND #20 |

# Supplementary Table 2: The quality assessment of Cross-Sectional Study.

| first author/year | 1. Define the source of information (survey, record review). | 2. List inclusion and exclusion criteria for exposed and unexposed subjects (cases and controls) or refer to previous publications. | 3. Indicate time period used for identifying patients. | 4. Indicate whether or High riskt subjects were consecutive if High riskt population-based. | 5. Indicate if evaluators of subjective components of study were masked to other aspects of the participants. | 6. Describe any assessments undertaken for quality assurance purposes (e.g.,test/retest of primary outcome measurements). | 7. Explain any patient exclusions from analysis. | 8. Describe how confounding was assessed and/or controlled. | 9. If applicable, explain how missing data were handled in the analysis. | 10. Summarize patient response rates and completeness of data collection. | 11. Clarify what follow-up, if any, was expected and the percentage of patients for which incomplete data or follow-up was obtained. | Overall |
| --- | --- | --- | --- | --- | --- | --- | --- | --- | --- | --- | --- | --- |
| Abdallah,2024 | Low risk | Low risk | Low risk | Low risk | Unclear | Unclear | Low risk | Low risk | High risk | High risk | High risk | Medium |
| Adelosoye,2020 | Low risk | Low risk | Low risk | Low risk | Low risk | Low risk | High risk | Low risk | High risk | Low risk | High risk | High |
| Al-Asadi,2015 | Low risk | Low risk | Low risk | Low risk | Unclear | Unclear | High risk | Low risk | High risk | High risk | High risk | Medium |
| Alhassan,2014 | Low risk | Low risk | Low risk | Low risk | Unclear | Unclear | High risk | Low risk | High risk | High risk | High risk | Medium |
| Anh,2023 | Low risk | Low risk | Low risk | Low risk | Unclear | Unclear | High risk | High risk | High risk | High risk | High risk | Medium |
| Beyene,2025 | Low risk | Low risk | Low risk | Low risk | Unclear | Unclear | High risk | Low risk | High risk | Low risk | High risk | Medium |
| Cai,2024 | Low risk | Low risk | Low risk | Low risk | Unclear | Unclear | High risk | Low risk | High risk | High risk | High risk | Medium |
| Chen,2013 | Low risk | Low risk | Low risk | Low risk | Unclear | Low risk | High risk | Low risk | High risk | High risk | High risk | Medium |
| Chen,2016 | Low risk | Low risk | Low risk | Low risk | Unclear | Low risk | High risk | Low risk | High risk | Low risk | High risk | Medium |
| Chen,2019 | Low risk | Low risk | Low risk | Low risk | Low risk | Low risk | High risk | Low risk | High risk | Low risk | High risk | High |
| Cheng,2011 | Low risk | Low risk | Low risk | Low risk | Unclear | Low risk | High risk | Low risk | High risk | Low risk | High risk | Medium |
| Cui,2021 | Low risk | Low risk | Low risk | Low risk | Unclear | Unclear | High risk | Low risk | High risk | Low risk | High risk | Medium |
| Deng,2018 | Low risk | Low risk | Low risk | Low risk | Unclear | Unclear | High risk | Low risk | High risk | High risk | High risk | Medium |
| Drosdzol,2009 | Low risk | Low risk | Low risk | Low risk | Unclear | Unclear | Low risk | Low risk | High risk | High risk | High risk | Medium |
| Fang,2015 | Low risk | Low risk | Low risk | Low risk | Unclear | Unclear | High risk | High risk | High risk | High risk | High risk | Medium |
| Guo,2017 | Low risk | Low risk | Low risk | Low risk | Unclear | Unclear | High risk | Low risk | High risk | High risk | High risk | Medium |
| Han,2013 | Low risk | Low risk | Low risk | Low risk | Unclear | Unclear | High risk | Low risk | High risk | High risk | High risk | Medium |
| Hao,2017 | Low risk | Low risk | Low risk | Low risk | Unclear | Unclear | High risk | High risk | High risk | High risk | High risk | Medium |
| Hasan,2023 | Low risk | High risk | Low risk | Low risk | Unclear | Unclear | High risk | Low risk | High risk | High risk | High risk | Medium |
| He,2014 | Low risk | Low risk | Low risk | Low risk | Unclear | Unclear | High risk | Low risk | High risk | High risk | High risk | Medium |
| High riskël,2022 | Low risk | Low risk | Low risk | Low risk | Unclear | Unclear | High risk | Low risk | High risk | High risk | High risk | Medium |
| Hu,2017 | Low risk | Low risk | Low risk | Low risk | Unclear | Unclear | High risk | Low risk | High risk | Low risk | High risk | Medium |
| Hu,2021 | Low risk | Low risk | Low risk | Low risk | Unclear | Low risk | High risk | Low risk | High risk | High risk | High risk | Medium |
| Joelsson,2017 | Low risk | Low risk | Low risk | Low risk | Unclear | Unclear | High risk | High risk | High risk | Low risk | High risk | Medium |
| Kumar,2024 | Low risk | High risk | Low risk | Low risk | Unclear | Unclear | High risk | Low risk | High risk | High risk | High risk | Medium |
| Lakatos,2017 | Low risk | Low risk | Low risk | Low risk | Unclear | Unclear | High risk | Low risk | High risk | Low risk | High risk | Medium |
| Leenakshi,2023 | Low risk | Low risk | Low risk | Low risk | Unclear | Unclear | High risk | High risk | High risk | High risk | High risk | Medium |
| Li,2012 | Low risk | Low risk | Low risk | Low risk | Unclear | Unclear | High risk | Low risk | High risk | High risk | High risk | Medium |
| Li,2016 | Low risk | Low risk | Low risk | Low risk | Unclear | Unclear | High risk | Low risk | High risk | High risk | High risk | Medium |
| Li,2017 | Low risk | Low risk | Low risk | Low risk | Unclear | Unclear | High risk | Low risk | High risk | High risk | High risk | Medium |
| Liang,2018 | Low risk | Low risk | Low risk | Low risk | Low risk | Low risk | High risk | Low risk | High risk | High risk | High risk | Medium |
| Liu,2010 | Low risk | High risk | Low risk | Low risk | Unclear | Unclear | High risk | High risk | High risk | High risk | High risk | Low |
| Ma,2022 | Low risk | Low risk | Low risk | Low risk | Unclear | Unclear | High risk | Low risk | High risk | High risk | High risk | Medium |
| Makanjuola,2010 | Low risk | High risk | Low risk | Low risk | Unclear | Unclear | Low risk | Low risk | High risk | High risk | High risk | Medium |
| Men,2020 | Low risk | Low risk | Low risk | Low risk | Unclear | Unclear | High risk | Low risk | High risk | High risk | High risk | Medium |
| Niazi,2024 | Low risk | Low risk | Low risk | Low risk | Unclear | Unclear | High risk | Low risk | High risk | High risk | High risk | Medium |
| Oladeji,2018 | Low risk | Low risk | Low risk | Low risk | Unclear | Unclear | High risk | Low risk | High risk | High risk | High risk | Medium |
| Peng,2019 | Low risk | Low risk | Low risk | Low risk | Unclear | Low risk | High risk | Low risk | High risk | Low risk | High risk | Medium |
| Qi,2007 | Low risk | High risk | Low risk | Low risk | Unclear | Unclear | High risk | High risk | High risk | High risk | High risk | Low |
| Ramezanzadeh,2004 | Low risk | Low risk | Low risk | Low risk | Unclear | Unclear | High risk | Low risk | High risk | High risk | High risk | Medium |
| Rufai,2022 | Low risk | Low risk | Low risk | Low risk | Unclear | Unclear | High risk | Low risk | High risk | High risk | High risk | Medium |
| Sezgin,2016 | Low risk | Low risk | Low risk | Low risk | Unclear | Unclear | High risk | Low risk | High risk | High risk | High risk | Medium |
| Sulyman,2018 | Low risk | Low risk | Low risk | Low risk | Unclear | Unclear | High risk | Low risk | High risk | High risk | High risk | Medium |
| Sun,2021 | Low risk | Low risk | Low risk | Low risk | Unclear | Low risk | High risk | Low risk | High risk | Low risk | High risk | Medium |
| Tan,2023 | Low risk | Low risk | Low risk | Low risk | Unclear | Unclear | High risk | Low risk | High risk | High risk | High risk | Medium |
| Tuan M Vo,2019 | Low risk | Low risk | Low risk | Low risk | Unclear | Unclear | High risk | Low risk | High risk | High risk | High risk | Medium |
| Wang,2014 | Low risk | Low risk | Low risk | Low risk | Unclear | Unclear | High risk | Low risk | High risk | High risk | High risk | Medium |
| Wang,2015 | Low risk | Low risk | Low risk | Low risk | Unclear | Low risk | High risk | Low risk | High risk | Low risk | High risk | Medium |
| Wang,2020 | Low risk | Low risk | Low risk | Low risk | Unclear | Unclear | High risk | Low risk | High risk | High risk | High risk | Medium |
| Wu,2008 | Low risk | High risk | Low risk | Low risk | Unclear | Unclear | High risk | Low risk | High risk | High risk | High risk | Medium |
| Wu,2014 | Low risk | High risk | Low risk | Low risk | Unclear | Unclear | High risk | High risk | High risk | High risk | High risk | Low |
| Wu,2015 | Low risk | Low risk | Low risk | Low risk | Unclear | Unclear | High risk | High risk | High risk | High risk | High risk | Medium |
| Yang,2023 | Low risk | Low risk | Low risk | Low risk | Unclear | Unclear | High risk | Low risk | High risk | High risk | High risk | Medium |
| Yusuf,2016 | Low risk | High risk | Low risk | Low risk | Unclear | Unclear | High risk | High risk | High risk | High risk | High risk | Low |
| Zhang,2013 | Low risk | Low risk | Low risk | Low risk | Unclear | Low risk | High risk | Low risk | Low risk | Low risk | High risk | High |
| Zhang,2017 | Low risk | Low risk | Low risk | Low risk | Low risk | Unclear | High risk | Low risk | High risk | Low risk | High risk | Medium |
| Zhang,2022 | Low risk | Low risk | Low risk | Low risk | Unclear | Unclear | Low risk | Low risk | High risk | High risk | High risk | Medium |
| Zhao,2018 | Low risk | Low risk | Low risk | Low risk | Unclear | Low risk | High risk | Low risk | High risk | Low risk | High risk | Medium |
| Zhou,2005 | Low risk | High risk | Low risk | Low risk | Unclear | Unclear | High risk | Low risk | High risk | High risk | High risk | Medium |
| Zhu,2022 | Low risk | Low risk | Low risk | Low risk | Unclear | Low risk | High risk | Low risk | High risk | Low risk | High risk | Medium |

# Supplementary Table 3: The quality assessment of Cross-Sectional Study.

| first author/year | SELECTION | | | | Definition of Controls | EXPOSURE | | | Overall |
| --- | --- | --- | --- | --- | --- | --- | --- | --- | --- |
|  | Is the Case Definition Adequate? | Representativeness of the Cases | Selection of Controls | Definition of Controls | Comparability of Cases and Controls on the Basis of the Design or Analysis | Ascertainment of Exposure | Same method of ascertainment for cases and controls | Non-Response Rate |  |
| - | a) yes, with independent validation b) yes, eg record linkage or based on self reports  c) no description | a) consecutive or obviously representative series of cases b) potential for selection biases or not stated | a) community controls b) hospital controls c) no description | a) no history of disease (endpoint)  b) no description of source | a) study controls for ___ (Select the most important factor.)  b) study controls for any additional factor (This criteria could be modified to indicate specific control for a second important factor.) | a) secure record (eg surgical records)  b) structured interview where blind to case/control status  c) interview not blinded to case/control status  d) written self report or medical record only  e) no description | a) yes  b) no | a) same rate for both groups  b) non respondents described  c) rate different and no designation | - |
| Ma,2018 | a | a | b | a | a | a | a | b | High |

**Supplementary Table 4: Comprehensive comparison of all psychometric instruments**

| **Instruments** | **Build dimensions** | **Number of items** | **Standard threshold** |
| --- | --- | --- | --- |
| HADS | anxiety、depression | 14 | ≥8 |
| DASS-21 | anxiety、depression | 21 | anxiety≥ 8; depression≥ 10 |
| SAS | anxiety、depression | 20 | ≥50 |
| SDS | depression | 20 | ≥53 |
| PHQ-9 | depression | 9 | ≥10 |
| BDI | depression | 21 | ≥14 |

# Supplementary Figures

# Supplementary Figures 1: Other Factors Contributing to Anxiety


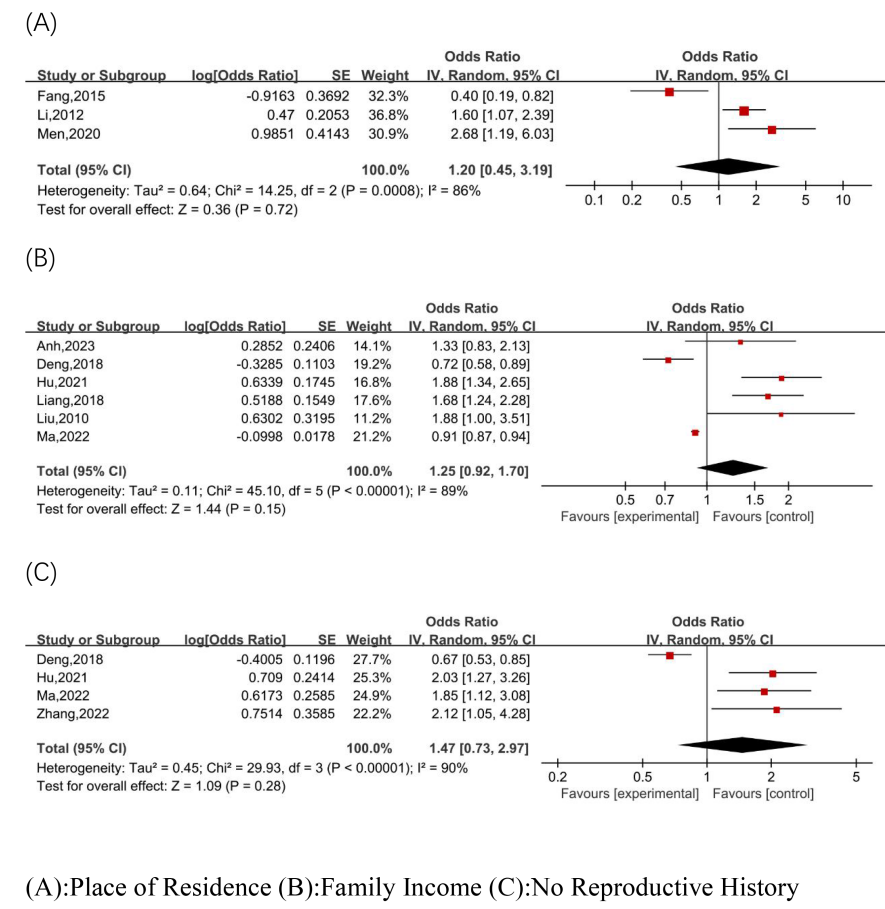


# Supplementary Figures 2: Other Factors Contributing to Depression


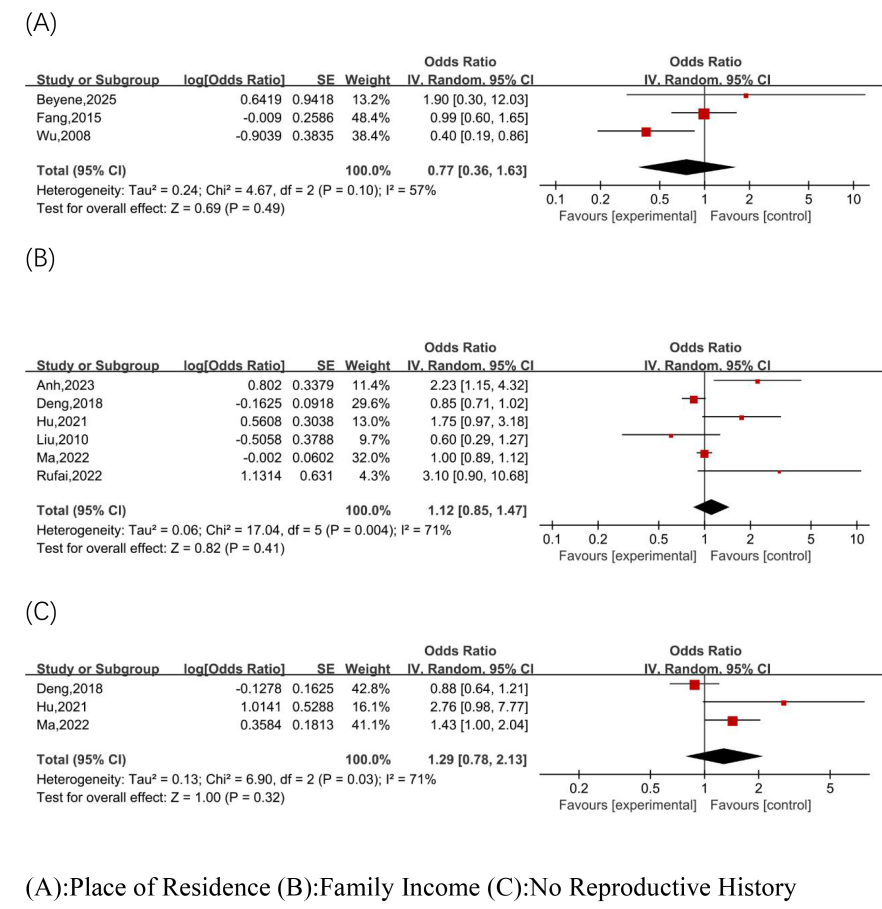

Supplement: Supplementary file 1 [file Supplementary_file_1.docx]
